# Discovery of Novel Indoleamine 2,3-Dioxygenase 1 (IDO1) and Histone Deacetylase 1 (HDAC1) Dual Inhibitors Derived from the Natural Product Saprorthoquinone

**DOI:** 10.3390/molecules25194494

**Published:** 2020-09-30

**Authors:** Yang Lin, Heyanhao Zhang, Tong Niu, Mei-Lin Tang, Jun Chang

**Affiliations:** 1 School of Pharmacy, Human Phenome Institute, Fudan University, Shanghai 201203, China; 14211030004@fudan.edu.cn (Y.L.); 17211030076@fudan.edu.cn (H.Z.); 18211030065@fudan.edu.cn (T.N.); 2 State Key Laboratory of Molecular Engineering and Institutes of Biomedical Sciences, Fudan University, Shanghai 200433, China

**Keywords:** saprorthoquinone, structural modification, IDO1, HDAC, dual inhibitors, antitumor

## Abstract

The discovery of IDO1 and HDAC1 dual inhibitors may provide a novel strategy for cancer treatment by taking advantages of both immunotherapeutic and epigenetic drugs. In this paper, saprorthoquinone (**1**) and 13 of its analogues from *Salvia prionitis* Hance were investigated for their SAR against IDO1, the results demonstrated the *ortho*-quinone was a key pharmacophore. Then a series of IDO1 and HDAC dual inhibitors connected by appropriate linkers were designed, synthesized, and evaluated from the hit compound saprorthoquinone (**1**). Among them, compound **33d** showed balanced activity against both IDO1 (IC_50_ = 0.73 μM) and HDAC1 (IC_50_ = 0.46 μM). Importantly, the structure of **33d** suggested that an *ortho*-quinone pharmacophore and a *N*-(2-aminophenyl) amide pharmacophore were necessary for the IDO inhibition and HDAC inhibition respectively. Meanwhile, these two pharmacophore groups should be combined by a pentane linker. Moreover, the binding modes of **33d** to the enzyme active site showed that the hydrogen bond with Leu234 of IDO1 appeared to confer increased potency to this class of inhibitors, which may explain the higher activity of **33d**. This study provides a new strategy for future IDO1/HDAC dual inhibitors with synergistic antitumor activity started from lead compound **33d**.

## 1. Introduction

Cancer is one of the most important factors have been seriously threatening human’s life and health. According to the GLOBOCAN 2018 estimates of cancer incidence and mortality produced by the International Agency for Research on Cancer, there were an estimated 18.1 million new cases of cancer and 9.6 million deaths from cancer worldwide in 2018 [1]. Traditional medications for cancer patients through targeting the tumor cells directly, showed a series of disadvantages such as low efficacy, severe side effects, muti-drug resistance, as well as significant individual differences [2]. On the contrary, cancer immunotherapy, which is one of the most promising treatments, has been an alternative fundamental strategy of cancer treatment by activating T cells to enhance patients’ native immune response [3]. The immune checkpoint inhibitors cytotoxic T-lymphocyte-associated protein 4 (CTLA-4) antibody ipilimumab and programmed death (PD-1) antibodies pembrolizumab and nivolumab were approved by FDA [4]. However, these biologic therapies exhibited inevitable weaknesses, such as risk of infection, high cost, and the requirement for intravenous injections [5]. By contrast, small-molecules for cancer immunotherapy with significant advantages, such as oral administration, access to intracellular targets, and greater drug exposure within the tumor microenvironment, which not only serves as a supplement for the monoclonal antibody, but also plays a synergistic effect, having important clinical and economic value [6]. Therefore, there is an urgent demand for developing small molecules to enhance the immune system and fight against cancer.

IDO1 is an intracellular heme-containing enzyme that catalyzes the oxidation of L-tryptophan (Trp) to *N*-formyl-L-kynurenine (Kyn) [7]. This enzyme is overexpressed in the microenvironment of several tumor types including carcinomas of the endometrium, cervix, kidney, lung, and colon [8]. In detail, IDO1 activity has a dual effect on the immune tolerogenic environment, including a depletion of Trp storages and an accumulation of Kyn products [9]. By co-opting IDO1 activity, tumor cells can mask their rapid cellular growth effectively and evade the host immune response [10]. Given the important role in tumor immune escape, IDO1 represents a valuable therapeutic target in cancer immunotherapy. Unfortunately, single treatments of IDO inhibitors showed limited antitumor activity in pre-clinical models. IDO inhibitor has been shown to behave synergistically when used in combination with therapies which target immune checkpoints [11]. Though the synergistic effects observed by the combination of IDO1 inhibitors with other therapies, drug combination strategies are always limited by complex pharmacokinetics and drug−drug interactions [12]. To overcome the problem, designing a single agent that simultaneously targets two or more synergistic mechanisms has attracted great interests [13].

**Figure 1:**
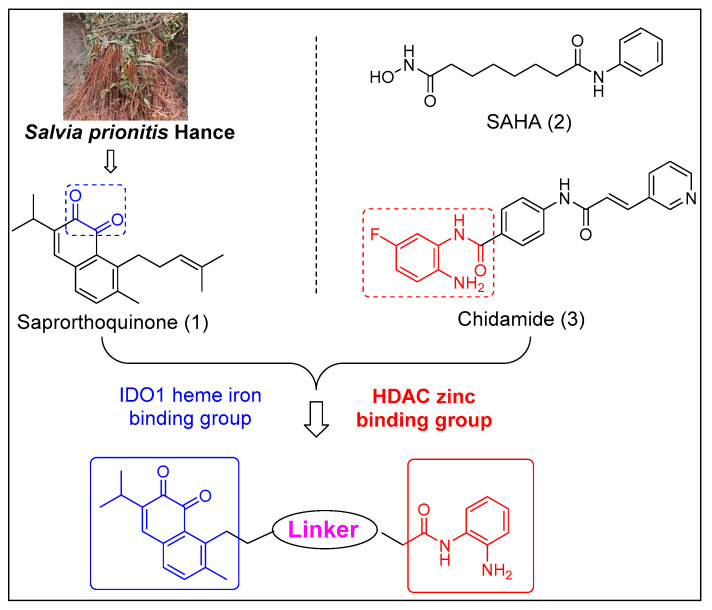
Chemical structures of IDO1 inhibitors and HDAC inhibitors, and design strategy of IDO1 and HDAC dual inhibitors.

Histone deacetylases (HDACs) are a family of enzymes, which catalyze the deacetylation of the lysine residues at the amino terminal of histones [14]. DNA deregulation and post-translational modifications of the histone tails is a characteristic feature of mammalian cancer. It had been seen that histone deacetylation causes tumor invasion and metastasis [15]. Studies resulted that HDAC inhibitors enhance the anticancer effectiveness of already present therapeutically and would be very influent in the co-activity together with other anticancer treatment approaches [16]. Several HDAC inhibitors, such as SAHA and chidamide, have been approved for the treatment of various hematological malignancies (Figure 1) [17]. Recent studies showed that HDACs could also improve tumor recognition and reverse immune suppression via various mechanisms [18]. Therefore, the discovery of dual IDO1 and HDAC inhibitors may provide a novel strategy for cancer treatment by taking advantages of both immunotherapeutic and epigenetic drugs.

Traditional Chinese Medicine (TCM) has evolved over thousands of years and is an invaluable source of inspiration for drug discovery, which led us to isolate its active components as lead compounds for drug development [19]. Some of the best IDO1 inhibitors are natural products that contain an oligocyclicquinone scaffold [20]. Saprorthoquinone was isolated from a traditional Chinese medicine *Salvia prionitis* Hance, and more than 40 diterpenoidal compounds have been isolated from this plant [21]. In 1999, Zhang reported that saprorthoquinone and its analogues showcased potent cytotoxicity [22]. Among them a novel compound named salvicine possessed a significant activity against malignant tumors, especially solid tumors such as lung cancer by inhibiting the topoisomerase II [23]. Furthermore, the preliminary structure-activity relationship of saprorthoquinone analogues was discussed by Deng in 2010 [24]. These compounds drawn our great interest due to their structural diversity. Consequently, we hope to obtain new breakthroughs by biological screening of these natural products to find a privileged scaffold different from those existed IDO1 inhibitors, which is valuable for further drug discovery and development.

**Figure 2:**
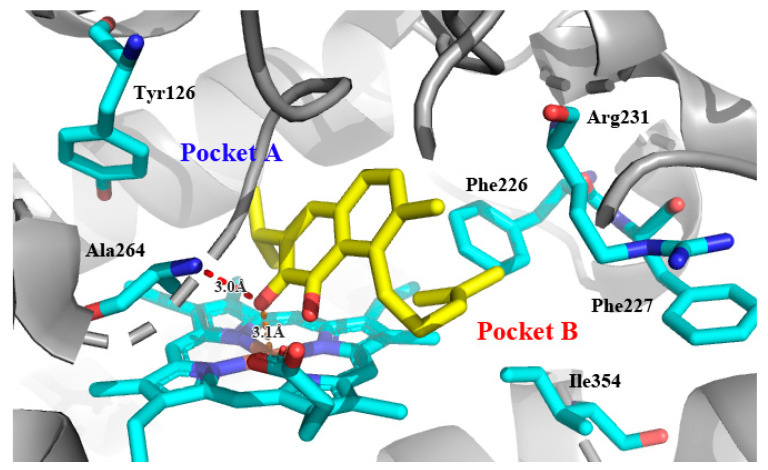
Modeling structure of **1**-IDO1 complex. Hydrogen bonds are labeled as red dashed lines, and coordination bonds are labeled as orange dashed lines. The figure was generated using PyMol.

Recently, we screened the IDO1 inhibitory activities of saprorthoquinone (**1**) and its analogues (Table 1). Surprisingly we found that *ortho*-quinones showed significantly better inhibitory activities than *para*-quinones among these compounds, while *para*-quinones were usually established as potent IDO1 inhibitors [20]. Among these compounds, **1** has both good activity and structural plasticity. To elucidate the binding mode of compound **1** to IDO1, we performed molecular docking. Predicted binding mode demonstrate that the carbonyl group of quinone scaffold formed hydrogen bonding interaction with Ala264 and coordination bonding interaction with Fe^2+^ of heme. The quinone scaffold of compound **1** fitted into one of IDO1 hydrophobic pockets (pocket A), and its side chain extended toward the direction of the other hydrophobic pocket (pocket B) that was mainly composed of Phe226, Phe227, Arg231 and Ile354 (Figure 2).

Based on above work, we used saprorthoquinone (**1**) as a hit compound and tried to find more effective IDO1 and HDAC dual inhibitors through modification of the side chain of the hit compound by a pharmacophore fusion strategy. Meanwhile, the zinc binding functional group *N*-(2-aminophenyl) amide (Chidamide as a template) is essential for HDAC inhibition can be introduced on the IDO heme binding scaffold directly or via a proper spacer (Figure 1). As a result, a series of novel IDO1 and HDAC dual targeting molecules were designed, synthesized, and assayed.

## 2. Results and Discussion

### 2.1. IDO1 Inhibitory Activities of Saprorthoquinone and Its Analogues Isolated from Salvia Prionitis Hance

Some of the best IDO1 inhibitors are natural products that contain an oligocyclic quinone scaffold [20]. Saprorthoquinone was isolated from a traditional Chinese medicine *Salvia prionitis* Hance, and more than 40 diterpenoid compounds have been isolated from this plant [21]. Recently, we screened the IDO1 inhibitory activities of saprorthoquinone (**1**) and its analogues, and the IDO1 inhibitor epacadostat (INCB024360) was chosen as positive controls (Table 1). Surprisingly we found that *ortho*-quinones showed significantly better inhibitory activities than *para*-quinones among these compounds, while *para*-quinones were usually established as potent IDO1 inhibitors. Among them, *ortho*-quinone analogues **1**, **4–9** exhibited over 60% IDO1 inhibitory activity in 10 μM. On the contrary, *para-*quinones analogues **10**–**16** exhibited lower than 30% IDO1 inhibitory activity in 10 μM. Some of them (**10**, **11**, **15**, and **16**) almost lost their activity. This result demonstrated that the *ortho*-quinone is the key pharmacophore in response to IDO1. Particularly, compound **1** and **7** exhibited the most potent IDO1 inhibitory activity (85% and 86%) at the concentration of 10 μM, which used for further drug discovery and development.

### 2.2. Novel Saprorthoquinone Analogues with Different Side Chain as Linker

Compound **1** with potent IDO1 inhibitory was chosen for structure-activity relationship study, in which the key pharmacophore *ortho-*quinone of **1** was maintained, and the side chain was transformed into three kinds of functional groups, such as amino group, phenylamino group and sulfonyl group.

**Scheme 1:**
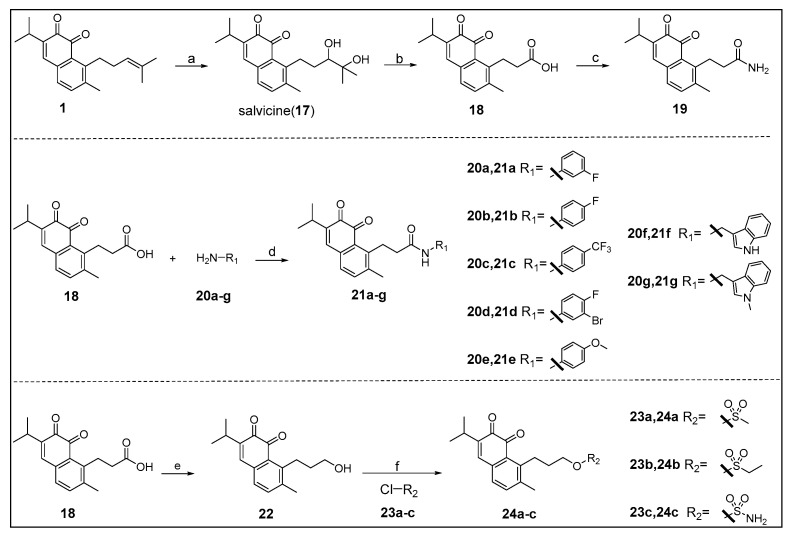
*Reagents and conditions*: (**a**) (I) *m*-CPBA, DCM, rt; (II) 8% perchloric acid, THF/H_2_O, rt, 92%; (**b**) PCC, H_2_O, DCM, rt, 47%; (**c**) (I) SOCl_2_, DCM, reflux; (II) NH_3_·H_2_O, DCM, 0 °C, 40%; (**d**) HATU, DIPEA, dry DMF, rt, 26–85%; (**e**) LiAlH_4_, dry THF, 0 °C, 53%; (**f**) dry DCM, 0 °C, 18–40%.

The key intermediate **18** was synthesized by the method reported in the literature (Scheme 1) [22]. Starting from the key intermediate, a simple amidation product **19** was prepared from an acid chloride. Then we used condensation reaction to get compounds **21a**–**g**, which allows for the rapid and versatile generation of different groups in the side chain, in order to allow for the penetration in the pocket B of IDO1 active site. Furthermore, an unstable compound **22** was obtained by reduction reaction, and then we synthesized stable sulfonyl products **24a**–**c**.

The activity of the first batch of products were assayed against IDO1 and A549 cell line with 1 and NLG-919 as the positive control and the results were shown in Table 2. Basically, when the side chain was replaced by a large group (phenyl), the IDO1 inhibitory activity was generally reduced (e.g., **21a**–**g**). On the contrary, if the side chain was replaced by a small group, the activity of IDO1 inhibitory could be increased. Sulfonyl group product **24a** showcased better IDO1 inhibitory activity than hit compound **1** (IC_50_ = 0.72 μM vs. IC_50_ = 1.76 μM). Amidated product **19** with a simple side chain gave the best enzymatic inhibitory activity (IC_50_ = 0.51 μM).

On the other hand, observation of cytotoxicities presented for these compounds couldn’t infer clear structure-activity relationship. Probably as reported in the literature, the cytotoxicities might be related to the ability of quinone carbonyl to accept one or two electrons to form the corresponding radical anion or dianion species [24].

### 2.3. IDO1 and HDAC Dual Inhibitors Derived from Natural Product Saprorthoquinone

**Scheme 2:**
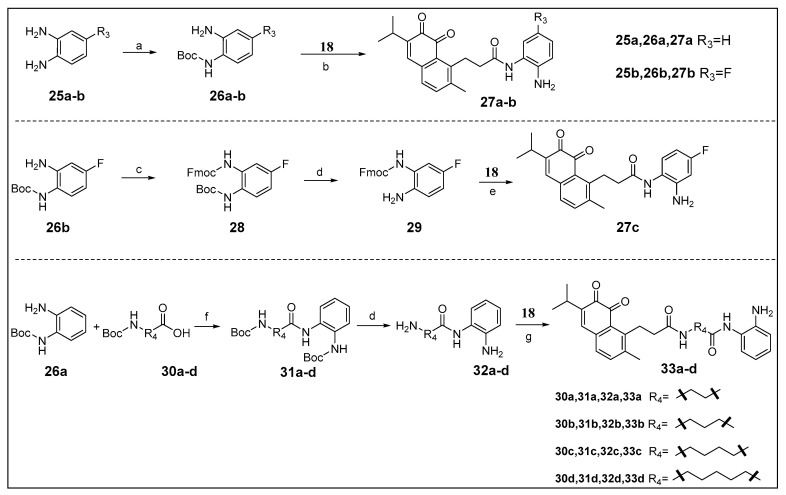
*Reagents and conditions*: (**a**) (Boc)_2_O, TEA, DCM, 0 °C, 66–76%; (**b**) (I) HATU, DIPEA, dry DMF, rt; (II) TFA, DCM, 0 °C, 10–27%; (**c**) Fmoc-Cl, NaHCO_3_, DCM, rt, 36%; (**d**) TFA, DCM, 0 °C; (**e**) (I) HATU, DIPEA, dry DMF, rt; (II) DEA, DCM, rt, 4%; (**f**) HATU, DIPEA, dry DMF, rt, 41–84%; (**g**) **18**, EDCI, DMAP, DIPEA, dry DMF, rt, 9–25%.

The zinc binding functional group *N*-(2-aminophenyl) amide is essential for HDAC inhibition can be introduced on the IDO1 heme binding scaffold directly or via a proper spacer (Scheme 2). As a result, a series of novel IDO1 and HDAC dual target molecules were designed, synthesized, and assayed. Compounds **27a** to **27c** were prepared to observe the effect of substituents on the phenyl ring, and **33a** to **33d** were used to observe the effect of the length of linker. Compounds **33a**–**d** were condensed by **32a**–**d** and **18** using EDCI as a condensing agent, because HATU is more advantageous for the condensation of an amine group on the phenyl ring. All products were purified by column chromatography.

The activities of the second batch of products were assayed against IDO1, HDAC1 and A549 cell line were shown in Table 3. NLG-919 and SAHA were used as the positive controls. The results showed that the *ortho*-substitution on the phenyl ring in the terminal amide group **27a** might have a positive effect on inhibitory activity of IDO1 (IC_50_ = 0.72 μM), the substitution of fluorine atoms at positions 4 (**27b**, 84% inhibiton @ 10 μM) and 5 (**27c**, 56% inhibiton @ 10 μM) confirmed the adverse effects of *meta* and *para*-substitution on IDO1 inhibition. Additionally, these three compounds (**27a**–**c**) have no inhibitory activity on HDAC1, due to their unsuitable linker. By contrast, the order of influence of carbon chain length on HDAC1 inhibition was five carbons (**33d**, IC_50_ = 0.46 μM) > three carbons (**33b**, IC_50_ = 34.87 μM) ≈ four carbons (**33c**, IC_50_ = 39.48 μM) > two carbons (**33a**, IC_50_ > 100 μM), compound **33d** was the most potent HDAC1 inhibitor. Notably, improvement of the IDO1 inhibitory was observed for these compounds with a long linker. However, compounds **33a**–**d** had reduced activity on the solid tumor cell line A549 may be due to the fact that IDO1 inhibitors do not destroy tumor cells directly, which may provide an indirect evidence for the increase of its inhibitory activity of IDO1. In vivo antitumor activity of compound **33d** is in the process experiment.

Besides, we have evaluated the toxicity of all the compounds on a non-tumor cell (ECs, primary endothelial cells). The cytotoxic concentration 50 (CC_50_) of all the compounds were over 200 μM. The results demonstrated the compounds have good safety.

### 2.4. Molecular Modeling Studies of Promising Compound ***1***, ***19*** and ***33d***

To gain insight into the molecular determinants that modulate the inhibitory activity of our compounds, molecular modeling studies were performed based on the X-ray cocrystal structure of IDO1 complex and HDAC1 complex. Protein structures were reconstructed by SWISS-MODEL homology-modelling server [25] using IDO1 (PDB code: 5EK2) and HDAC1 (PDB code: 4BKX) as templates. Compound **1**, **19** and **33d** were selected as the ligands for the molecular docking. Since the high flexibility of side chain and backbone of IDO1 protein, classical semi-flexible docking method often poses a problem for IDO1 docking. To overcome difficulty mentioned above, after docking by AutoDock software, best poses of **33d**-protein complexes with dominant conformation underwent QM/MM simulations [26].

**Figure 3:**
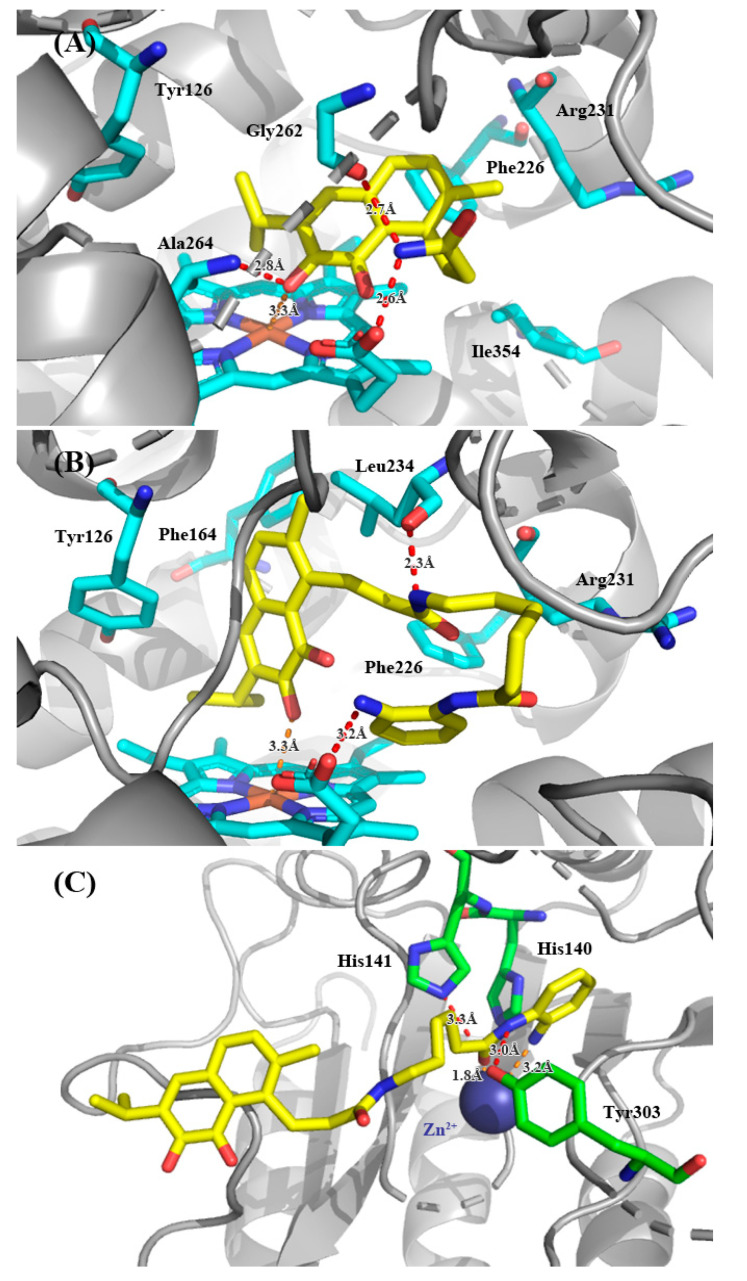
Modeling structure of ligand-protein complexes. (**A**,**B**) For compound **19** and **33d** with IDO1. (**C**) For compound **33d** with HDAC1. Hydrogen bonds are labeled as red dashed lines, and coordination bonds are labeled as orange dashed lines. The figure was generated using PyMol.

Docking poses of the most promising compounds (**19** and **33d**) and human IDO1 are reported in Figure 3A,B. Docking studies showing that these analogues of saprorthoquinone had similar binding modes to compound **1** (Figure 2). The quinone scaffold of both **19** and **33d** was accommodated in the hydrophobic pocket A (Mainly composed of Tyr126, Phe163, Phe164, Gly262, and Ala264), while the carbonyl group of quinone scaffold was able to form a strong coordination bond with the heme group. The carbonyl group of quinone scaffold could also formed hydrogen bonding interactions with Ala264, which is same as the binding mode of 1. The NH group on the amide or terminal amine group could form a hydrogen bond with the oxygen atom on heme, Gly262 or Leu234, which is different from the predicted binding mode of compound **1**. Probably for this reason, compounds **19** and **33d** could bind to pocket A more strongly and had higher inhibitory activities against IDO1. However, none of these compounds had a good combination with amino-acid residue of pocket B (Phe226, Arg231 and Ile354). The linker in the structure may be very important for occupying of pocket B, and further optimization of such inhibitors in ongoing in our lab.

For the binding mode of **33d** with HDAC1, main interactions were involved in the zinc binding group (ZBG) (Figure 3C). Docking studies were carried out using the same method as above. Besides chelating with Zn^2+^, the *N*-(2-aminophenyl) amide group was engaged with a hydrogen bonding network with His141 and Tyr303. The amide NH group formed hydrogen bonding interaction with the phenolic hydroxyl group of Tyr303, and the carbonyl group of ZBG formed hydrogen bond with the nitrogen atom in the imidazole of His141.

To evaluate the dynamics stabilities of the complexes of compound **33d** with IDO1 and HDAC1, GROMACS software [27] with charm 36 forcefield [28] was applied to conduct the simulations. Complexes and proteins in the apo form (no ligand bound) were simulated under the temperature of 310 K and pressure of 1 bar for 100 ns. Sodium or chloride ions were added to the water-protein systems to preserve electroneutrality.

**Figure 4:**
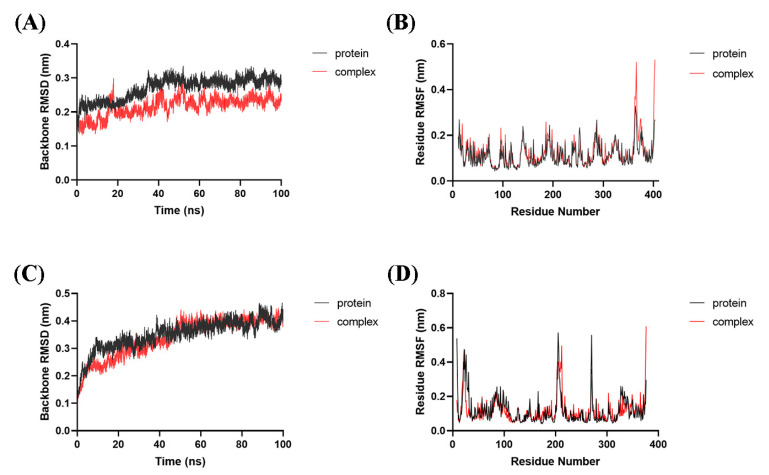
Dynamic simulations of **33d** bound with IDO1 or HDAC1. RMSD analysis of IDO1 (**A**) and HDAC1 (**C**) *in apo* or bounding form. RMSF analysis of IDO1 (**B**) and HDAC1 (**D**) *in apo* or bounding form.

All models went stable in the later stage of the simulations. The backbone atomic RMSD values of IDO1 and HDAC1 proteins *in apo* and bounding forms with **33d** were 0.292 ± 0.013 nm (IDO1 *in apo*), 0.233 ± 0.012 nm (IDO1 bounding form), 0.398 ± 0.022 nm (HDAC1 *in apo*) and 0.402 ± 0.014 nm (HDAC1 bounding form) during the last 30 ns of simulation, respectively (Figure 4A,C). These results indicated that the presence of **33d** bound to IDO1 and HDAC1 influenced the backbone atomic RMSD. The ligand-bound proteins exhibited the similar or less mobility than proteins *in apo* state under the same simulation conditions.

Similar RMSF distributions were observed for the IDO1 and HDAC1 *in apo*/bounding forms, respectively (Figure 4B,D). The greatest fluctuations observed in the terminals of IDO1/HDAC1 and regions (residues 203–212 and 265–274) of HDAC1. Residues in Pocket A and Pocket B of IDO1, including Tyr126, Phe163, Phe164, Phe226, Arg231, Gly262 and Ala264, were quite stable. Residues in active sites of HDAC1, such as His140, His141 and Tyr303, were stable too. Overall, the RMSF analysis indicated that the complex and protein exhibited similar dynamics during the MD simulations.

## 3. Materials and Methods

### 3.1. Chemistry

#### 3.1.1. General Information

All starting materials were commercially available and used without further purification unless otherwise stated. TLC analysis was performed using pre-coated glass plates. Column chromatography was performed using silica gel (300–400 mesh) or octadecylsilyl silica gel (75 μm) for separation and purification. ^1^H-NMR and ^13^C-NMR spectra were recorded on DRX-400 spectrometer (Bruker, Billerica, MA, USA) with CDCl_3_, CD_3_OD, acetone-*d*_6_ or DMSO-*d*_6_ as solvents. Resonances (*δ*) are given in parts per million relatives to tetramethylsilane or a residual solvent peak (CDCl_3_: ^1^H: *δ* = 7.26 ppm, ^13^C: *δ* = 77.00 ppm; CD_3_OD: ^1^H: *δ* = 3.31 ppm, ^13^C: *δ* = 49.00 ppm; acetone-*d*_6_: ^1^H: *δ* = 2.05 ppm, ^13^C: *δ* = 206.26 ppm, 29.84 ppm; DMSO-*d*_6_: ^1^H: *δ* = 2.50 ppm, ^13^C: *δ* = 39.50 ppm). Data are reported as follows: chemical shift; multiplicity (s = singlet, d = doublet, t = triplet, q = quartet, m = multiplet); coupling constants (Hz); and integration. HRMS were obtained on an Triple TOF^®®^ 5600^+^ (AB Sciex, Foster City, CA, USA). More spectral information can be found in Supplementary Materials.

#### 3.1.2. Synthesis of 8-(3,4-Dihydroxy-4-methylpentyl)-3-isopropyl-7-methylnaphthalene-1,2-dione (**17**)

To a stirred solution of *m*-chloroperbenzoic acid (1.45 g, 8.40 mmol) in dichloromethane (24 mL) held at 0 °C was added a solution of compound **1** (2 g, 6.75 mmol) dissolved in dichloromethane (20 mL) during a period of 30 min. The mixture was stirred overnight at room temperature, washed with saturated solution of NaHCO_3_ and dried over anhydrous MgSO_4_. The solvent was concentrated *in vacuo* and dissolved in tetrahydrofuran (35 mL) was added 6.7 mL of water. The solution was stirred and 4 mL of 8% perchloric acid was added. After stirring for 6 h under N_2_ at room temperature, saturated solution of NaCl was added and the mixture was extracted several times with ethyl acetate. The organic phase was washed with dilute sodium bicarbonate and dried over anhydrous MgSO_4_, evaporated under reduced pressure and purified by silica gel column chromatography using petroleum ether/ethyl acetate mixture (4:1 to 1:1) as eluent to afford **17** as an orange solid in 92% yield (2.05 g). ^1^H-NMR (400 MHz, CDCl_3_) *δ* 7.38 (d, *J* = 7.6 Hz, 1H, Ar-H), 7.10 (s, 1H, Ar-H), 7.09 (d, *J* = 7.6 Hz, 1H, Ar-H), 3.36–3.27 (m, 1H, CH of hydroxymethine), 3.15–3.05 (m, 1H, one of Ar-CH_2_ protons), 3.05–2.99 (m, 1H, CH of isopropyl group), 2.98–2.93 (m, 1H, one of Ar-CH_2_ protons), 2.39 (s, 3H, Ar-CH_3_), 1.90–1.78 (m, 1H, one of CH_2_CO protons), 1.75–1.64 (m, 1H, one of CH_2_CO protons), 1.33 (s, 3H, CH_3_), 1.28 (s, 3H, CH_3_), 1.18 (d, *J* = 6.9 Hz, 3H, CH_3_ of isopropyl group), 1.17 (d, *J* = 6.9 Hz, 3H, CH_3_ of isopropyl group). ESI-MS *m*/*z*: 353.2 [M + Na]^+^.

#### 3.1.3. Synthesis of 3-(6-Isopropyl-2-methyl-7,8-dioxo-7,8-dihydronaphthalen-1-yl)propanoic acid (**18**)

Compound **17** (505.2 mg, 1.53 mmol) was dissolved in dichloromethane (5 mL), pyridiniumchlorochromate (3.5 g, 16.24 mmol) was added and 1.5 mL of water was added dropwise. The mixture was stirred overnight at room temperature. The crude material was filtered through kieselguhr and purified by silica gel column chromatography using petroleum ether/ethyl acetate mixture (3:1 to 1:2) as eluent to afford **18** as an orange solid in 47% yield (204.9 mg). ^1^H-NMR (400 MHz, CDCl_3_) *δ* 7.38 (d, *J* = 7.6 Hz, 1H, Ar-H), 7.09 (s, 1H, Ar-H), 7.08 (d, *J* = 7.6 Hz, 1H, Ar-H), 3.35 (t, *J* = 7.7 Hz, 2H, Ar-CH_2_), 3.00 (septet, *J* = 6.9 Hz, 1H, CH), 2.61 (t, *J* = 7.7 Hz, 2H, CH_2_CO), 2.39 (s, 3H, Ar-CH_3_), 1.15 (d, *J* = 6.9 Hz, 6H, 2CH_3_). ESI-MS *m*/*z*: 287.0 [M + H]^+^.

#### 3.1.4. Synthesis of 3-(6-Isopropyl-2-methyl-7,8-dioxo-7,8-dihydro- naphthalen-1-yl)propanamide (**19**)

To a solution of compound **18** (100.0 mg, 0.35 mmol) in dichloromethane (5 mL) held at 0 °C was added thionylchloride (100 μL) and 1*H*-benzotriazole (42.9 mg, 0.36 mmol), and the mixture was stirred at reflux temperature for 2 h. The solvent was concentrated *in vacuo* and dissolved in dichloromethane (1 mL). Ammonium hydroxide solution was added at 0 °C and the mixture was stirred at room temperature for 1 h. Water was added and the mixture was extracted several times with DCM. The organic phase was dried over anhydrous MgSO_4_, evaporated under reduced pressure and purified by octadecylsilyl silica gel column chromatography using methanol/water (2:3) as eluent to afford **19** as an orange solid in 40% yield (40.0 mg). ^1^H NMR (400 MHz, CDCl_3_) *δ* 7.41 (d, *J* = 7.1 Hz, 1H, Ar-H), 7.12 (d, *J* = 7.1 Hz, 2H, Ar-H), 6.39 (s, 1H, one of CONH_2_ protons), 5.45 (s, 1H, one of CONH_2_ protons), 3.33 (m, 2H, Ar-CH_2_), 3.03 (septet, *J* = 5.9 Hz, 1H, CH), 2.43 (s, 3H, Ar-CH_3_), 2.39 (m, 2H, CH_2_CO), 1.18 (d, *J* = 5.9 Hz, 6H, 2CH_3_). ^13^C NMR (100 MHz, CDCl_3_) *δ* 182.67, 181.09, 174.87, 146.50, 144.98, 140.32, 140.26, 137.47, 135.31, 128.87, 128.14, 35.27, 27.88, 26.99, 21.48, 19.74. ESI-MS *m*/*z*: 308.0 [M + Na]^+^. HRESI-MS calcd. for C_17_H_20_NO_3_^+^ ([M + H]^+^) 286.1438, found 286.1440.

#### 3.1.5. General Procedure A: Synthesis of Compounds **21a**–**21g** and **31a**–**31d**

Compound **18** or **30a**–**30d** was dissolved in 1 mL of dry *N*,*N*-*dimethylformamide, 2-(7-aza-1H-benzotriazole-1-yl)-1,1,3,3-tetramethyluronium hexafluorophosphate* (HATU) and *N-ethyl-diisopropylamine* (DIPEA) were added. After stirring uniformly, amine **20a**–**20g** or **26a** was added. The mixture was stirred overnight at room temperature. dichloromethane was added and the mixture was washed with saturated solution of NaHCO_3_,the organic phase was purified by column chromatography to afford **21a**–**21g** or **31a**–**31d**.

*N-(3-Fluorophenyl)-3-(6-isopropyl-2-methyl-7,8-dioxo-7,8-dihydronaphthalen-1-yl)propanamide* (**21a**): Compound **18** (40.1 mg, 0.14 mmol), HATU (64.6 mg, 0.17 mmol), DIPEA (45.3 μL, 0.26 mmol) and **20a** (16.3 μL, 0.17 mmol) were used in general procedure A. The crude product was purified by octadecylsilyl silica gel column chromatography using methanol/water (3:1) as eluent to afford **21a** as an orange solid in 72% yield (38.4 mg). ^1^H-NMR (400 MHz, CDCl_3_) *δ* 8.42 (s, 1H, CONH), 7.64 (d, *J* = 11.3 Hz, 1H, Ar-H of phenyl group), 7.44 (d, *J* = 7.5 Hz, 1H, Ar-H of naphthoquinone), 7.31–7.22 (m, 2H, Ar-H of phenyl group), 7.15–7.10 (m, 2H, Ar-H of naphthoquinone), 6.88–6.71 (m, 1H, Ar-H of phenyl group), 3.42–3.32 (m, 2H, Ar-CH_2_), 3.03 (septet, *J* = 6.9 Hz, 1H, CH), 2.55–2.48 (m, 2H, CH_2_CO), 2.45 (s, 3H, Ar-CH_3_), 1.19 (d, *J* = 6.9 Hz, 6H, 2CH_3_).^13^C-NMR (100 MHz, CDCl_3_) *δ* 182.76, 181.04, 170.93, 162.99 (d, *J* = 244.3 Hz), 146.31, 144.96, 140.38, 140.32, 139.74 (d, *J* = 10.9 Hz), 137.64, 135.40, 129.94 (d, *J* = 9.3 Hz), 129.02, 128.11, 115.11, 110.71 (d, *J* = 21.3 Hz), 107.29 (d, *J* = 26.4 Hz), 36.96, 27.83, 27.06, 21.45, 19.75. ESI-MS *m*/*z*: 380.0 [M + H]^+^. HRESI-MS calcd. for C_23_H_23_FNO_3_^+^ ([M + H]^+^) 380.1656, found 380.1659.

*N-(4-Fluorophenyl)-3-(6-isopropyl-2-methyl-7,8-dioxo-7,8-dihydronaphthalen-1-yl)propanamide* (**21b**): Compound **18** (28.6 mg, 0.10 mmol), HATU (49.4 mg, 0.13 mmol), DIPEA (34.8 μL, 0.20 mmol) and **20b** (12.3 μL, 0.13 mmol) were used in general procedure A. The crude product was purified by octadecylsilyl silica gel column chromatography using methanol/water (3:1) as eluent to afford **21b** as an orange solid in 53% yield (20.1 mg). ^1^H-NMR (400 MHz, CDCl_3_) *δ*8.25 (s, 1H, CONH), 7.63–7.56 (m, 2H, Ar-H of phenyl group), 7.43 (d, *J* = 7.7 Hz, 1H, Ar-H of naphthoquinone), 7.12 (s, 1H, Ar-H of naphthoquinone), 7.12 (d, *J* = 7.7 Hz, 1H, Ar-H of naphthoquinone), 7.06–6.99 (m, 2H, Ar-H of phenyl group), 3.43–3.34 (m, 2H, Ar-CH_2_), 3.03 (septet, *J* = 6.9 Hz, 1H, CH), 2.55–2.45 (m, 2H, CH_2_CO), 2.45 (s, 3H, Ar-CH_3_), 1.19 (d, *J* = 6.9 Hz, 6H, 2CH_3_). ^13^C-NMR (100 MHz, CDCl_3_) *δ* 182.82, 181.08, 170.70, 159.30 (d, *J* = 243.3 Hz), 146.37, 145.02, 140.36, 140.24, 137.59, 135.41, 134.17, 128.95, 128.15, 121.75 (d, *J* = 7.8 Hz), 115.51 (d, *J* = 22.4 Hz), 36.88, 27.97, 27.08, 21.44, 19.71. ESI-MS *m*/*z*: 380.0 [M + H]^+^. HRESI-MS calcd. for C_23_H_23_FNO_3_^+^ ([M + H]^+^) 380.1656, found 380.1660.

*3-(6-Isopropyl-2-methyl-7,8-dioxo-7,8-dihydronaphthalen-1-yl)-N-(4-(trifluoromethyl)phenyl)propanamide* (**21c**): Compound **18** (28.6 mg, 0.10 mmol), HATU (49.4 mg, 0.13 mmol), DIPEA (34.8 μL, 0.20 mmol) and **20c** (20.9 mg, 0.13 mmol) were used in general procedure A. The crude product was purified by octadecylsilyl silica gel column chromatography using methanol/water (3:1) as eluent to afford **21c** as an orange solid in 85% yield (36.5 mg). ^1^H-NMR (400 MHz, CDCl_3_) *δ* 8.58 (s, 1H, CONH), 7.79 (d, *J* = 8.3 Hz, 2H, Ar-H of phenyl group), 7.59 (d, *J* = 8.3 Hz, 2H, Ar-H of phenyl group), 7.44 (d, *J* = 7.6 Hz, 1H, Ar-H of naphthoquinone), 7.14 (d, *J* = 7.6 Hz, 1H, Ar-H of naphthoquinone), 7.13 (s, 1H, Ar-H of naphthoquinone), 3.43–3.34 (m, 2H, Ar-CH_2_), 3.03 (septet, *J* = 6.8 Hz, 1H, CH), 2.57–2.48 (m, 2H, CH_2_CO), 2.45 (s, 3H, Ar-CH_3_), 1.19 (d, *J* = 6.8 Hz, 6H, 2CH_3_). ^13^C-NMR (100 MHz, CDCl_3_) *δ* 182.82, 181.05, 171.16, 146.26, 144.97, 141.36, 140.36, 137.70, 135.43, 129.07, 128.10, 126.15 (q, *J* = 3.4 Hz), 125.69 (q, *J* = 32.1 Hz), 122.84, 119.42, 36.95, 27.76, 27.07, 21.44, 19.74. ESI-MS *m*/*z*: 430.0 [M + H]^+^. HRESI-MS calcd. for C_24_H_23_F_3_NO_3_^+^ ([M + H]^+^) 430.1625, found 430.1625.

*N-(3-Bromo-4-fluorophenyl)-3-(6-isopropyl-2-methyl-7,8-dioxo-7,8-dihydronaphthalen-1-yl)propanamide* (**21d**): Compound **18** (40.1 mg, 0.14 mmol), HATU (64.6 mg, 0.17 mmol), DIPEA (45.3 μL, 0.26 mmol) and **20d** (32.1 mg, 0.17 mmol) were used in general procedure A. The crude product was purified by octadecylsilyl silica gel column chromatography using methanol/water (4:1) as eluent to afford **21d** as an orange solid in 53% yield (34.0 mg).^1^H-NMR (400 MHz, CDCl_3_) *δ* 8.33 (s, 1H, CONH), 8.02–7.95 (m, 1H, Ar-H of phenyl group), 7.56–7.49 (m, 1H, Ar-H of phenyl group), 7.45 (d, *J* = 7.7 Hz, 1H, Ar-H of naphthoquinone), 7.14 (d, *J* = 7.7 Hz, 1H, Ar-H of naphthoquinone), 7.13 (s, 1H, Ar-H of naphthoquinone), 7.12–7.05 (m, 1H, Ar-H of phenyl group), 3.41–3.33 (m, 2H, Ar-CH_2_), 3.04 (septet, *J* = 6.9 Hz, 1H, CH), 2.52–2.47 (m, 2H, CH_2_CO), 2.45 (s, 3H, Ar-CH_3_), 1.19 (d, *J* = 6.9 Hz, 6H, 2CH_3_). ^13^C-NMR (100 MHz, DMSO-*d*_6_) *δ* 182.11, 180.85, 171.27, 154.46 (d, *J* = 242.1 Hz), 145.79, 144.13, 140.46, 140.44, 137.16, 135.15, 134.20 (d, *J* = 12.4 Hz), 129.33, 128.96, 123.68, 120.44 (d, *J* = 6.7 Hz), 117.14 (d, *J* = 22.8 Hz), 107.95 (d, *J* = 21.6 Hz), 35.47, 26.96, 25.74, 21.79, 19.84. ESI-MS *m*/*z*: 458.8 [M + H]^+^. HRESI-MS calcd. for C_23_H_22_BrFNO_3_^+^ ([M + H]^+^) 458.0762, found 458.0765.

*3-(6-Isopropyl-2-methyl-7,8-dioxo-7,8-dihydronaphthalen-1-yl)-N-(4-methoxyphenyl)propanamide* (**21e**): Compound **18** (28.6 mg, 0.10 mmol), HATU (49.4 mg, 0.13 mmol), DIPEA (34.8 μL, 0.20 mmol) and **20e** (16.0 mg, 0.13 mmol) were used in general procedure A. The crude product was purified by octadecylsilyl silica gel column chromatography using methanol/water (3:1) as eluent to afford **21e** as an orange solid in 26% yield (10.3 mg).^1^H-NMR (400 MHz, CDCl_3_) *δ* 8.06 (s, 1H, CONH), 7.52 (d, *J* = 8.8 Hz, 2H, Ar-H of phenyl group), 7.43 (d, *J* = 7.7 Hz, 1H, Ar-H of naphthoquinone), 7.13 (s, 1H, Ar-H of naphthoquinone), 7.12 (d, *J* = 7.7 Hz, 1H, Ar-H of naphthoquinone), 6.88 (d, *J* = 8.8 Hz, 2H, Ar-H of phenyl group), 3.80 (s, 3H, OCH_3_), 3.44–3.35 (m, 2H, Ar-CH_2_), 3.04 (septet, *J* = 6.9 Hz, 1H, CH), 2.52–2.47 (m, 2H, CH_2_CO), 2.45 (s, 3H, Ar-CH_3_), 1.19 (d, *J* = 6.9 Hz, 6H, 2CH_3_). ^13^C-NMR (100 MHz, CDCl_3_) *δ* 182.75, 181.11, 170.51, 156.34, 146.52, 145.00, 140.41, 140.25, 137.54, 135.36, 131.26, 128.90, 128.16, 121.85, 114.11, 55.49, 36.90, 28.11, 27.05, 21.47, 19.78. ESI-MS *m*/*z*: 392.0 [M + H]^+^. HRESI-MS calcd. for C_24_H_26_NO_4_^+^ ([M + H]^+^) 392.1856, found 392.1861.

*N-((1H-indol-3-yl)methyl)-3-(6-isopropyl-2-methyl-7,8-dioxo-7,8-dihydronaphthalen-1-yl)propanamide* (**21f**): Compound **18** (28.6 mg, 0.10 mmol), HATU (49.4 mg, 0.13 mmol), DIPEA (34.8 μL, 0.20 mmol) and **20f** (19.0 mg, 0.13 mmol) were used in general procedure A. The crude product was purified by flash chromatography on silica gel then octadecylsilyl silica gel column chromatography using methanol/water (3:1) as eluent to afford **21f** as an orange solid in 52% yield (21.5 mg). ^1^H-NMR (400 MHz, CDCl_3_) *δ* 8.36 (s, 1H, CONH), 7.65 (d, *J* = 7.8 Hz, 1H, Ar-H of indole), 7.37 (d, *J* = 7.8 Hz, 1H, Ar-H of naphthoquinone), 7.23–7.01 (m, 5H, Ar-H, 3H of indole and 2H of naphthoquinone), 6.45–6.36 (m, 1H, Ar-H of indole), 4.67 (d, *J* = 5.3 Hz, 2H, CH_2_ by indole), 3.34–3.26 (m, 2H, CH_2_ bynaphthoquinone), 2.99 (septet, *J* = 6.9Hz, 1H, CH), 2.42 (s, 3H, Ar-CH_3_), 2.41–2.31 (m, 2H, CH_2_CO), 1.15 (d, *J* = 6.9 Hz, 6H, 2CH_3_). ^13^C-NMR (100 MHz, CDCl_3_) *δ* 182.39, 181.08, 172.29, 146.66, 144.79, 140.50, 140.37, 137.35, 136.41, 135.12, 128.76, 128.13, 126.59, 123.27, 122.30, 119.76, 118.99, 112.94, 111.31, 35.67, 35.19, 27.79, 26.95, 21.47, 19.84. ESI-MS *m*/*z*: 415.2 [M + H]^+^. HRESI-MS calcd. for C_26_H_27_N_2_O_3_^+^ ([M + H]^+^) 415.2016, found 415.2026.

*3-(6-Isopropyl-2-methyl-7,8-dioxo-7,8-dihydronaphthalen-1-yl)-N-((1-methyl-1H-indol-3-yl)methyl)-propanamide* (**21g**): Compound **18** (28.6 mg, 0.10 mmol), HATU (49.4 mg, 0.13 mmol), DIPEA (34.8 μL, 0.20 mmol) and **20g** (20.8 mg, 0.13 mmol) were used in general procedure A. The crude product was purified by flash chromatography on silica gel then octadecylsilyl silica gel column chromatography using methanol/water (4:1) as eluent to afford **21g** as an orange solid in 44% yield (19.0 mg). ^1^H-NMR (400 MHz, CDCl_3_) *δ* 7.65 (d, *J* = 7.9 Hz, 1H, Ar-H of indole), 7.37 (d, *J* = 7.6 Hz, 1H, Ar-H of naphthoquinone), 7.32–7.04 (m, 6H, CONH and 3Ar-H of indole and 2Ar-H of naphthoquinone), 6.40–6.29 (m, 1H, Ar-H of indole), 4.65 (d, *J* = 5.2 Hz, 2H, CH_2_ by indole), 3.77 (s, 3H, NCH_3_), 3.34–3.26 (m, 2H, CH_2_ by naphthoquinone), 3.00 (septet, *J* = 6.9 Hz, 1H, CH), 2.43 (s, 3H, Ar-CH_3_), 2.39–2.31 (m, 2H, CH_2_CO), 1.15 (d, *J* = 6.9 Hz, 6H, 2CH_3_). ^13^C-NMR (100 MHz, CDCl_3_)*δ* 182.42, 181.08, 172.17, 146.65, 144.81, 140.48, 140.28, 137.32, 137.14, 135.13, 128.73, 128.16, 127.93, 127.06, 121.89, 119.33, 119.12, 111.44, 109.32, 35.73, 35.03, 32.73, 27.82, 26.95, 21.47, 19.84. ESI-MS *m*/*z*: 429.2 [M + H]^+^. HRESI-MS calcd. for C_27_H_29_N_2_O_3_^+^ ([M + H]^+^) 429.2173, found 429.2175.

*tert-Butyl-(3-((2-((tert-butoxycarbonyl)amino)phenyl)amino)-3-oxopropyl)carbamate* (**31a**): Compound **30a** (39.7 mg, 0.21 mmol), HATU (95.1 mg, 0.25 mmol), DIPEA (73.2 μL, 0.42 mmol) and **26a** (52.0 mg, 0.25 mmol) were used in general procedure A. The crude product was purified by silica gel column chromatography using petroleum ether/ethyl acetate mixture (3:1 to 1:1) as eluent to afford **31a** as a colorless oil in 84% yield (66.5 mg). ^1^H-NMR (400 MHz, CDCl_3_) *δ*8.59 (s, 1H,Ph-NHCO), 7.54–7.44 (m, 1H, Ar-H), 7.38 (d, *J* = 7.0 Hz, 1H, Ar-H), 7.25 (s, 1H, Ph-NHCO), 7.21–7.06 (m, 2H, Ar-H), 5.32 (s, 1H, CONH), 3.58–3.29 (m, 2H, NH-CH_2_), 2.64–2.36 (m, 2H, CH_2_CO), 1.51 (s, 9H, 3CH_3_), 1.43 (s, 9H, 3CH_3_).ESI-MS *m*/*z*: 402.2 [M + Na]^+^.

*tert-Butyl-(2-(4-((tert-butoxycarbonyl)amino)butanamido)phenyl)carbamate* (**31b**): Compound **30b** (99.5 mg, 0.49 mmol), HATU (224.2 mg, 0.59 mmol), DIPEA (170.7 μL, 0.98 mmol) and **26a** (122.8 mg, 0.59 mmol) were used in general procedure A. The crude product was purified by octadecylsilyl silica gel column chromatography using methanol/water (3:2) as eluent to afford **31b** as a colorless oil in 41% yield (79.2 mg). ^1^H-NMR (400 MHz, CDCl_3_) *δ*8.98 (s, 1H,Ph-NHCO), 7.57 (d, *J* = 7.0 Hz, 1H, Ar-H), 7.47 (s, 1H,Ph-NHCO), 7.35 (d, *J* = 7.0 Hz, 1H, Ar-H), 7.15 (t, *J* = 7.0 Hz, 1H, Ar-H), 7.07 (t, *J* = 7.0 Hz, 1H, Ar-H), 5.03 (s, 1H, CONH), 3.21–3.08 (m, 2H, NH-CH_2_), 2.37–2.26 (m, 2H, CH_2_CO), 1.87–1.75 (m, 2H, CH_2_), 1.50 (s, 9H, 3CH_3_), 1.45 (s, 9H, 3CH_3_). *m*/*z*: 416.2 [M + Na]^+^.

*tert-Butyl(2-(5-((tert-butoxycarbonyl)amino)pentanamido)phenyl)carbamate (***31c**): Compound **30c** (99.9 mg, 0.46 mmol), HATU (209.1 mg, 0.55 mmol), DIPEA (160.2 μL, 0.92 mmol) and **26a** (114.5 mg, 0.55 mmol) were used in general procedure A. The crude product was purified by octadecylsilyl silica gel column chromatography using methanol/water (3:2) as eluent to afford **31c** as a colorless oil in 53% yield (99.0 mg).^1^H-NMR (400 MHz, CDCl_3_) *δ* 8.74 (s, 1H,Ph-NHCO), 7.50 (d, *J* = 7.5 Hz, 1H, Ar-H), 7.44 (s, 1H,Ph-NHCO), 7.30 (d, *J* = 7.5 Hz, 1H, Ar-H), 7.14 (t, *J* = 7.5 Hz, 1H, Ar-H), 7.06 (t, *J* = 7.5 Hz, 1H, Ar-H), 4.86 (s, 1H, CONH), 3.10–2.99 (m, 2H, NH-CH_2_), 2.36–2.21 (m, 2H, CH_2_CO), 1.70–1.56 (m, 2H, CH_2_), 1.50 (s, 9H, 3CH_3_), 1.47–1.33 (m, 11H, CH_2_ and 3CH_3_). ESI-MS *m*/*z*: 430.2 [M + Na]^+^.

*tert-Butyl(2-(6-((tert-butoxycarbonyl)amino)hexanamido)phenyl)carbamate* (**31d**): Compound **30d** (99.4 mg, 0.43 mmol), HATU (178.7 mg, 0.47 mmol), DIPEA (149.8 μL, 0.86 mmol) and **26a** (97.8 mg, 0.47 mmol) were used in general procedure A. The crude product was purified by octadecylsilyl silica gel column chromatography using methanol/water (3:2) as eluent to afford **31d** as a colorless oil in 50% yield (91.2 mg). ^1^H-NMR (400 MHz, CDCl_3_) *δ* 8.61 (s, 1H,Ph-NHCO), 7.45 (d, *J* = 7.3 Hz, 1H, Ar-H), 7.36 (d, *J* = 7.3 Hz, 1H, Ar-H), 7.35 (s, 1H,Ph-NHCO), 7.14 (t, *J* = 7.3 Hz, 1H, Ar-H), 7.08 (t, *J* = 7.3 Hz, 1H, Ar-H), 4.76 (s, 1H, CONH), 3.17–2.92 (m, 2H, NH-CH_2_), 2.46–2.16 (m, 2H, CH_2_CO), 1.75–1.57 (m, 2H, CH_2_), 1.51 (s, 9H, 3CH_3_), 1.47–1.37 (m, 11H, CH_2_ and 3CH_3_), 1.36–1.24 (m, 2H, CH_2_). ESI-MS *m*/*z*: 444.2 [M + Na]^+^.

#### 3.1.6. Synthesis of 8-(3-Hydroxypropyl)-3-isopropyl-7-methylnaphthalene-1,2-dione (22)

Compound **18** (80.1 mg, 0.28 mmol) was dissolved in 4 mL of dry tetrahydrofuran, and a solution of 1M lithium aluminum hydride in tetrahydrofuran (560 μL, 0.56 mmol) was added portionwise over a period of 30 min. After refluxing for 1 h, 24 μL of water was added dropwise, followed by an equal volume of 15% sodium hydroxide and 3 times the amount of water. The crude material was filtered through kieselguhr and purified by silica gel column chromatography using petroleum ether/ethyl acetate mixture (3:1 to 1:1) as eluent to afford **22** as an orange oil (40.7mg, 53% yield). ^1^H-NMR (400 MHz, CDCl_3_) *δ* 7.36 (d, *J* = 7.6 Hz, 1H, Ar-H), 7.08 (s, 1H, Ar-H), 7.05 (d, *J* = 7.6 Hz, 1H, Ar-H), 3.79 (t, *J* = 6.1 Hz, 2H, OH-CH_2_), 3.14–3.07 (m, 2H, Ar-CH_2_), 3.01 (septet, *J* = 6.9 Hz, 1H, CH), 2.38 (s, 3H, Ar-CH_3_), 1.80–1.71 (m, 2H, CH_2_), 1.15 (d, *J* = 6.9 Hz, 6H, 2CH_3_). ESI-MS *m*/*z*: 295.2 [M + Na]^+^.

#### 3.1.7. General Procedure B: Synthesis of Compounds **24a**–**24c**

To a solution of compound **22** (32.7 mg, 0.12 mmol) in dry dichloromethane (5 mL) held at 0 °C was added dry triethylamine (0.1 mL, 0.74 mmol) and **23a**–**23c**. The mixture was stirred 2 h at room temperature. Saturated solution of NaHCO_3_ was added and the mixture was extracted several times with dichloromethane. The organic phase was dried over anhydrous MgSO_4_, evaporated under reduced pressure and purified by octadecylsilyl silica gel column chromatography using methanol/water (7:3) as eluent to afford compounds **24a**–**24c**.

*3-(6-Isopropyl-2-methyl-7,8-dioxo-7,8-dihydronaphthalen-1-yl)propyl methanesulfonate* (**24a**): Compound **23a** (53.8 mg, 0.47 mmol) were used in general procedure B to afford **24a** as an orange solid in 38% yield (16.2 mg). ^1^H-NMR (400 MHz, CDCl_3_) *δ* 7.40 (d, *J* = 7.6 Hz, 1H, Ar-H), 7.11 (s, 1H, Ar-H), 7.10 (d, *J* = 7.6 Hz, 1H, Ar-H), 4.43 (t, *J* = 6.1 Hz, 2H, CH_2_OSO_2_), 3.18–3.10 (m, 2H, Ar-CH_2_), 3.07 (s, 3H, SO_2_CH_3_), 3.01 (septet, *J* = 6.8 Hz, 1H, CH), 2.39 (s, 3H, Ar-CH_3_), 2.02–1.91 (m, 2H, CH_2_), 1.17 (d, *J* = 6.8 Hz, 6H, 2CH_3_). ^13^C-NMR (100 MHz, CDCl_3_) *δ* 182.45, 181.18, 146.44, 144.92, 140.22, 140.13, 137.16, 135.16, 128.67, 128.35, 70.46, 37.38, 28.28, 26.95, 26.48, 21.48, 19.78. ESI-MS *m*/*z*: 373.0 [M + Na]^+^. HRESI-MS calcd. for C_18_H_22_NaO_5_S^+^ ([M + Na]^+^) 373.1080, found 373.1086.

*3-(6-Isopropyl-2-methyl-7,8-dioxo-7,8-dihydronaphthalen-1-yl)propyl ethanesulfonate* (**24b**): Compound **23b** (46.3 mg, 0.36 mmol) were used in general procedure B to afford **24b** as an orange solid in 18% yield (8.0 mg). ^1^H-NMR (400 MHz, CDCl_3_) *δ* 7.41 (d, *J* = 7.6 Hz, 1H, Ar-H), 7.11 (s, 1H, Ar-H), 7.10 (d, *J* = 7.6 Hz, 1H, Ar-H), 4.43 (t, *J* = 6.2 Hz, 2H, CH_2_OSO_2_), 3.19 (q, *J* = 7.4 Hz, 2H, SO_2_CH_2_), 3.16–3.11 (m, 2H, Ar-CH_2_), 3.03 (septet, *J* = 6.9 Hz, 1H, CH), 2.40 (s, 3H, Ar-CH_3_), 2.02–1.92 (m, 2H, CH_2_), 1.46 (t, *J* = 7.4 Hz, 3H, CH_3_), 1.17 (d, *J* = 6.9 Hz, 6H, 2CH_3_ of isopropyl group). ^13^C-NMR (100 MHz, CDCl_3_) *δ*182.41, 181.18, 146.49, 144.88, 140.24, 140.13, 137.14, 135.13, 128.66, 128.33, 70.13, 44.88, 28.41, 26.94, 26.51, 21.48, 19.77, 8.24. ESI-MS *m*/*z*: 387.0 [M + Na]^+^. HRESI-MS calcd. for C_19_H_24_NaO_5_S^+^ ([M + Na]^+^) 387.1237, found 387.1241.

*3-(6-Isopropyl-2-methyl-7,8-dioxo-7,8-dihydronaphthalen-1-yl)propyl sulfamate* (**24c**): Compound **23c** (32.7 mg, 0.12 mmol) were used in general procedure B to afford **24c** as an orange solid in 40% yield (17.0 mg). ^1^H-NMR (400 MHz, CD_3_OD) *δ* 7.48 (d, *J* = 7.9 Hz, 1H, Ar-H), 7.31 (s, 1H, Ar-H), 7.24 (d, *J* = 7.9 Hz, 1H, Ar-H), 4.28 (t, *J* = 6.3 Hz, 2H, CH_2_OSO_2_), 3.24–3.11 (m, 2H, Ar-CH_2_), 2.96 (septet, *J* = 6.9 Hz, 1H, CH), 2.41 (s, 3H, Ar-CH_3_), 2.00–1.84 (m, 2H, CH_2_), 1.18 (d, *J* = 6.9 Hz, 6H, 2CH_3_).^13^C-NMR (100 MHz, acetone-*d*_6_) *δ* 182.41, 181.01, 146.03, 144.42, 140.02, 139.90, 136.82, 135.32, 128.73, 69.78, 28.04, 26.87, 26.10, 20.82, 18.85. ESI-MS *m*/*z*: 374.0 [M + Na]^+^. HRESI-MS calcd. for C_17_H_21_NNaO_5_S^+^ ([M + Na]^+^) 374.1033, found 374.1038.

#### 3.1.8. General Procedure C: Synthesis of Compounds **26a**–**26b**

The reactant was dissolved in 6 mL of dichloromethane, and 0.1 mL of triethylamine was added. Di-tert-butyl dicarbonate was added at 0 °C and the mixture was stirred overnight at room temperature. The mixture was washed with water to give the crude product, which was used in the next reaction directly.

*tert-Butyl (2-aminophenyl)carbamate* (**26a**): Compound **25a** (198.8 mg, 1.84 mmol) was used in general procedure C to afford **26a** as a light yellow oil in 76% yield (292.5 mg). ESI-MS *m*/*z*: 231.0 [M + Na]^+^.

*tert-Butyl (2-amino-4-fluorophenyl)carbamate* (**26b**): Compound **25b** (99.6 mg, 0.79 mmol) was used in general procedure C to afford **26b** as a light yellow oil in 66% yield (117.6 mg). ESI-MS *m*/*z*: 249.0 [M + Na]^+^.

#### 3.1.9. General Procedure D: Synthesis of Compounds **27a**–**27b**

Compound **18** was dissolved in 1 mL of dry *N*,*N*-dimethylformamide, HATU and DIPEA were added. After stirring uniformly, amine **26a**–**26b** was added. The mixture was stirred overnight at room temperature. Dichloromethane was added and the mixture was washed with saturated solution of NaHCO_3_, the organic phase was purified by octadecylsilyl silica gel column chromatography using methanol/water as eluent. The solvent was concentrated in vacuo and dissolved in 2.5 mL dichloromethane, 1 mL of trifluoroacetic acid was added at 0 °C and the mixture was stirred 1 h at room temperature. Saturated solution of NaHCO_3_ was added and the mixture was extracted several times with dichloromethane. The organic phase was dried over anhydrous MgSO_4_, evaporated under reduced pressure to afford compound **27a**–**27b**.

*N-(2-Aminophenyl)-3-(6-isopropyl-2-methyl-7,8-dioxo-7,8-dihydronaphthalen-1-yl)propanamide* (**27a**): Compound **18** (40.1 mg, 0.14 mmol), HATU (64.6 mg, 0.17 mmol), DIPEA (45.3 μL, 0.26 mmol) and **26a** (33.3 mg, 0.16 mmol) were used in general procedure D. The crude product was purified by octadecylsilyl silica gel column chromatography using methanol/water (4:1) as eluent. Finally affording **27a** as an orange solid in 27% yield (14.1 mg). ^1^H-NMR (400 MHz, CDCl_3_) *δ* 8.04 (s, 1H, CONH), 7.43 (d, *J* = 7.6 Hz, 1H, Ar-H of phenyl group), 7.31 (d, *J* = 7.9 Hz, 1H, Ar-H of naphthoquinone), 7.12 (s, 1H, Ar-H of naphthoquinone), 7.12 (d, *J* = 7.9 Hz, 1H, Ar-H of naphthoquinone), 7.05 (t, *J* = 7.6 Hz, 1H, Ar-H of phenyl group), 6.84–6.75 (m, 2H, Ar-H of phenyl group), 3.49–3.37 (m, 2H, Ar-CH_2_), 3.02 (septet, *J* = 6.8 Hz, 1H, CH), 2.61–2.49 (m, 2H, CH_2_CO), 2.44 (s, 3H, Ar-CH_3_), 1.18 (d, *J* = 6.8 Hz, 6H, 2CH_3_). ^13^C-NMR (100 MHz, CDCl_3_) *δ* 182.75, 181.04, 171.18, 146.44, 144.99, 140.59, 140.39, 140.20, 137.51, 135.37, 128.91, 128.22, 126.95, 125.27, 124.31, 119.30, 117.83, 36.27, 27.98, 27.05, 21.47, 19.82. ESI-MS *m*/*z*: 377.2 [M + H]^+^. HRESI-MS calcd. for C_23_H_25_N_2_O_3_^+^ ([M + H]^+^) 377.1860, found 377.1865.

*N-(2-Amino-5-fluorophenyl)-3-(6-isopropyl-2-methyl-7,8-dioxo-7,8-dihydronaphthalen-1-yl)propanamide* (**27b**): Compound **18** (85.8 mg, 0.30 mmol), HATU (136.8 mg, 0.36 mmol), DIPEA (104.5 μL, 0.60 mmol) and **26b** (81.4 mg, 0.36 mmol) were used in general procedure D. The crude product was purified by octadecylsilyl silica gel column chromatography using methanol/water (4:1) as eluent. Finally affording **27b** as an orange solid in 10% yield (12.0 mg). ^1^H-NMR (400 MHz, CDCl_3_) *δ* 8.18 (s, 1H, CONH), 7.48–7.38 (m, 2H, Ar-H, 1H of phenyl group and 1H of naphthoquinone), 7.13 (d, *J* = 6.6 Hz, 1H, Ar-H of naphthoquinone), 7.12 (s, 1H, Ar-H of naphthoquinone), 6.79–6.72 (m, 2H, Ar-H of phenyl group), 3.46–3.34 (m, 2H, Ar-CH_2_), 3.02 (septet, *J* = 6.7 Hz, 1H, CH), 2.60–2.50 (m, 2H, CH_2_CO), 2.45 (s, 3H, Ar-CH_3_), 1.18 (d, *J* = 6.7 Hz, 6H, 2CH_3_). ^13^C-NMR (100 MHz, CDCl_3_) *δ* 182.84, 180.88, 171.03, 162.50 (d, *J* = 266.9 Hz), 146.36, 145.00, 140.34, 140.22, 137.64, 135.43, 134.77, 129.01, 128.16, 126.39 (d, *J* = 10.1 Hz), 118.83 (d, *J* = 8.6 Hz), 112.45 (d, *J* = 22.4 Hz), 110.83 (d, *J* = 26.0 Hz), 36.57, 28.02, 27.05, 21.48, 19.84. ESI-MS *m*/*z*: 395.2 [M + H]^+^. HRESI-MS calcd. for C_23_H_24_FN_2_O_3_^+^ ([M + H]^+^) 395.1765, found 395.1775.

#### 3.1.10. Synthesis of (9*H*-fluoren-9-yl) methyl *tert*-butyl (4-fluoro-1,2-phenylene)dicarbamate (**28**)

Compound **26b** (169.6 mg, 0.75 mmol) was dissolved in 3 mL of dichloromethane, and 6 mL of saturated solution of NaHCO_3_ was added. The mixture was stirred and (9*H*-fluoren-9-ylmethoxy)carbonyl chloride (271.6 mg, 1.05 mmol) was added. 3 h later, the mixture was washed by water and was extracted several times with dichloromethane. The organic phase was dried over anhydrous MgSO_4_, and purified by octadecylsilyl silica gel column chromatography using methanol/water (1:1) as eluent to afford **28** as a brown oil in 36% yield (121.1 mg). ESI-MS *m*/*z*: 471.0 [M + Na]^+^.

#### 3.1.11. General Procedure E: Synthesis of Compounds **29** and **32a–32d**

Compound **28** or **31a**–**31d** was dissolved in 2.5 mL dichloromethane, 1 mL of trifluoroacetic acid was added at 0 °C and the mixture was stirred 1 h at room temperature. Saturated solution of NaHCO_3_ was added and the mixture was extracted several times with dichloromethane. The organic phase was dried over anhydrous MgSO_4_, evaporated under reduced pressure to afford compound **29** and **32a**–**32d**, which was used in the next reaction directly.

#### 3.1.12. Synthesis of *N*-(2-amino-4-fluorophenyl)-3-(6-isopropyl-2-methyl-7,8-dioxo-7,8-dihydronaphthalen-1-yl)propanamide (**27c**)

Compound **18** (60.1 mg, 0.21 mmol) was dissolved in 1 mL of dry *N*,*N*-dimethylformamide, HATU (95.1 mg, 0.25 mmol) and DIPEA (73.2 μL, 0.42 mmol) were added. After stirring uniformly, **29** (88.5 mg, 0.25 mmol) was added. The mixture was stirred overnight at room temperature. Dichloromethane was added and the mixture was washed with saturated solution of NaHCO_3_, the organic phase was purified by silica gel column chromatography using petroleum ether/ethyl acetate mixture (4:1 to 1:1) as eluent. The solvent was concentrated in vacuo and dissolved in 2 mL dichloromethane, 2 mL diethylamine was added and the mixture was stirred 1 h at room temperature. The mixture was evaporated under reduced pressure and purified by octadecylsilyl silica gel column chromatography using methanol/water (3:2) as eluent to afford **27c** as an orange solid in 4% Yield (4.3 mg). ^1^H-NMR (400 MHz, CDCl_3_) *δ* 8.14 (s, 1H, CONH), 7.44 (d, *J* = 7.7 Hz, 1H, Ar-H of naphthoquinone), 7.26–7.22 (m, 1H, Ar-H of phenyl group), 7.13 (s, 1H, Ar-H of naphthoquinone), 7.13 (d, *J* = 7.7 Hz, 1H, Ar-H of naphthoquinone), 6.63–6.51 (m, 2H, Ar-H of phenyl group), 3.46–3.38 (m, 2H, Ar-CH_2_), 3.02 (septet, *J* = 6.8 Hz, 1H, CH), 2.60–2.52 (m, 2H, CH_2_CO), 2.45 (s, 3H, Ar-CH_3_), 1.18 (d, *J* = 6.8 Hz, 6H, 2CH_3_). ^13^C-NMR (100 MHz, CDCl_3_) *δ*182.83, 181.02, 171.48, 160.55 (d, *J* = 264.4 Hz), 146.38, 145.04, 142.93 (d, *J* = 12.2 Hz), 140.34, 140.23, 137.63, 135.44, 129.00, 128.16, 127.27 (d, *J* = 10.1 Hz), 119.65, 105.50 (d, *J* = 22.6 Hz), 103.91 (d, *J* = 25.6 Hz), 36.18, 28.14, 27.06, 21.48, 19.82. ESI-MS *m*/*z*: 395.0 [M + H]^+^. HRESI-MS calcd. for C_23_H_24_FN_2_O_3_^+^ ([M + H]^+^) 395.1765, found 395.1769.

#### 3.1.13. General Procedure F: Synthesis of Compounds **33a**–**33d**

Compound **18** was dissolved in 1 mL of dry *N*,*N*-dimethylformamide, 3-(3-dimethylaminopropyl)-1-ethylcarbodiimide hydrochloride (EDCI), 4-dimethylaminopyridine (DMAP) and DIPEA were added. After stirring uniformly, amine **32a–32d** was added. The mixture was stirred overnight at room temperature. Dichloromethane was added and the mixture was washed with saturated solution of NaHCO_3_,the organic phase was purified by column chromatography to afford **33a–33d**.

*N-(2-aminophenyl)-3-(3-(6-isopropyl-2-methyl-7,8-dioxo-7,8-dihydronaphthalen-1-yl)propanamido)-propanamide* (**33a**): Compound **18** (28.6 mg, 0.10 mmol), EDCI (23.0 mg, 0.12 mmol), DMAP (1 mg), DIPEA (104.5 μL, 0.60 mmol) and **32a** were used in general procedure F. The crude product was purified by flash chromatography on silica gel then octadecylsilyl silica gel column chromatography using methanol/water (3:2) as eluent to afford **33a** as an orange solid in 12% yield (5.7 mg).^1^H-NMR (400 MHz, CDCl_3_) *δ* 8.21 (s, 1H, Ph-NHCO), 7.38 (d, *J* = 7.4 Hz, 1H, Ar-H of phenyl group), 7.24 (d, *J* = 7.8 Hz, 1H, Ar-H of naphthoquinone), 7.09 (s, 1H, Ar-H of naphthoquinone), 7.08 (d, *J* = 7.8 Hz, 1H, Ar-H of naphthoquinone), 7.02 (t, *J* = 7.4 Hz, 1H, Ar-H of phenyl group), 6.90–6.83 (m, 1H, Ar-H of phenyl group), 6.81–6.70 (m, 2H, CONH and Ar-H of phenyl group), 3.71–3.59 (m, 2H, NH-CH_2_), 3.36–3.23 (m, 2H, Ar-CH_2_), 3.00 (septet, *J* = 6.2 Hz, 1H, CH), 2.71–2.61 (m, 2H, CH_2_CO), 2.38 (s, 3H, Ar-CH_3_), 2.37–2.29 (m, 2H, CH_2_CO), 1.17 (d, *J* = 6.2 Hz, 6H, 2CH_3_). ^13^C-NMR (100 MHz, CDCl_3_) *δ* 182.55, 181.20, 172.97, 170.37, 146.53, 144.77, 140.88, 140.55, 140.32, 137.32, 135.11, 128.82, 128.34, 127.10, 125.58, 124.07, 119.12, 117.82, 36.66, 35.79, 35.40, 27.11, 26.96, 21.48, 19.76. ESI-MS *m*/*z*: 448.2 [M + H]^+^. HRESI-MS calcd. for C_26_H_30_N_3_O_4_^+^ ([M + H]^+^) 448.2231, found 448.2238.

*N-(2-Aminophenyl)-4-(3-(6-isopropyl-2-methyl-7,8-dioxo-7,8-dihydronaphthalen-1-yl)propanamido)-butanamide* (**33b**): Compound **18** (51.5 mg, 0.18 mmol), EDCI (38.3 mg, 0.20 mmol), DMAP (1.7 mg), DIPEA (188.1 μL, 1.08 mmol) and **32b** were used in general procedure F. The crude product was purified by flash chromatography on silica gel then octadecylsilyl silica gel column chromatography using methanol/water (7:3) as eluent to afford **33b** as an orange solid in 11% yield (10.2 mg). ^1^H-NMR (400 MHz, CDCl_3_) *δ* 8.65 (s, 1H, Ph-NHCO), 7.42 (d, *J* = 7.5 Hz, 1H, Ar-H of phenyl group), 7.37 (d, *J* = 7.8 Hz, 1H, Ar-H of naphthoquinone), 7.12 (s, 1H, Ar-H of naphthoquinone), 7.11 (d, *J* = 7.8 Hz, 1H, Ar-H of naphthoquinone), 7.02 (t, *J* = 7.5 Hz, 1H, Ar-H of phenyl group), 6.82–6.70 (m, 3H, CONH and 2Ar-H of phenyl group), 3.53–3.44 (m, 2H, NH-CH_2_), 3.33–3.24 (m, 2H, Ar-CH_2_), 3.02 (septet, *J* = 6.9 Hz, 1H, CH), 2.51–2.45 (m, 2H, CH_2_CO), 2.41 (s, 3H, Ar-CH_3_), 2.39–2.32 (m, 2H, CH_2_CO), 2.03–1.93 (m, 2H, CH_2_), 1.17 (d, *J* = 6.9 Hz, 6H, 2CH_3_). ^13^C-NMR (100 MHz, CDCl_3_) *δ* 182.75, 181.06, 173.78, 171.45, 146.58, 144.93, 140.58, 140.39, 140.33, 137.56, 135.34, 128.94, 128.10, 126.62, 125.16, 124.54, 118.96, 117.64, 38.40, 35.83, 33.96, 28.07, 27.04, 26.79, 21.46, 19.75. ESI-MS *m*/*z*: 462.2 [M + H]^+^. HRESI-MS calcd. for C_27_H_32_N_3_O_4_^+^ ([M + H]^+^) 462.2387, found 462.2398.

*N-(2-Aminophenyl)-5-(3-(6-isopropyl-2-methyl-7,8-dioxo-7,8-dihydronaphthalen-1-yl)propanamido)-pentanamide* (**33c**): Compound **18** (57.2 mg, 0.20 mmol), EDCI (46.0 mg, 0.24 mmol), DMAP (1.9 mg), DIPEA (209.0 μL, 1.20 mmol) and **32c** were used in general procedure F. The crude product was purified by flash chromatography on silica gel then octadecylsilyl silica gel column chromatography using methanol/water (7:3) as eluent to afford **33c** as an orange solid in 9% yield (10.6 mg).^1^H-NMR (400 MHz, CDCl_3_) *δ* 7.83 (s, 1H, Ph-NHCO), 7.40 (d, *J* = 7.4 Hz, 1H, Ar-H of naphthoquinone), 7.11 (s, 1H, Ar-H of naphthoquinone), 7.09 (d, *J* = 7.4 Hz, 1H, Ar-H of naphthoquinone), 7.03 (t, *J* = 7.3 Hz, 1H, Ar-H of phenyl group), 6.83–6.53 (m, 3H, CONH and 2Ar-H of phenyl group), 3.43–3.36 (m, 2H, NH-CH_2_), 3.32–3.23 (m, 2H, Ar-CH_2_), 3.01 (septet, *J* = 6.8 Hz, 1H, CH), 2.56–2.47 (m, 2H, CH_2_CO), 2.38 (s, 3H, Ar-CH_3_), 2.36–2.29 (m, 2H, CH_2_CO), 1.89–1.79 (m, 2H, CH_2_), 1.74–1.63 (m, 2H, CH_2_), 1.17 (d, *J* = 6.8 Hz, 6H, 2CH_3_). ^13^C-NMR (100 MHz, DMSO-*d*_6_) *δ* 182.01, 180.86, 171.76, 171.48, 146.30, 144.02, 142.30, 140.53, 140.48, 137.06, 135.08, 129.19, 128.83, 126.12, 125.68, 124.02, 116.60, 116.33, 38.77, 35.89, 34.66, 29.28, 26.94, 26.16, 23.27, 21.79, 19.91. ESI-MS *m*/*z*: 476.2 [M + H]^+^. HRESI-MS calcd. for C_28_H_34_N_3_O_4_^+^ ([M + H]^+^) 476.2544, found 476.2548.

*N-(2-Aminophenyl)-6-(3-(6-isopropyl-2-methyl-7,8-dioxo-7,8-dihydronaphthalen-1-yl)propanamido) hexanamide* (**33d**): Compound **18** (57.2 mg, 0.20 mmol), EDCI (46.0 mg, 0.24 mmol), DMAP (1.9 mg), DIPEA (209.0 μL, 1.20 mmol) and **32d** were used in general procedure F. The crude product was purified by flash chromatography on silica gel then octadecylsilyl silica gel column chromatography using methanol/water (7:3) as eluent to afford **33d** as an orange solid in 25% yield (25.7 mg).^1^H-NMR (400 MHz, CDCl_3_) *δ* 7.97 (s, 1H, Ph-NHCO), 7.39 (d, *J* = 7.5 Hz, 1H, Ar-H of phenyl group), 7.19 (d, *J* = 7.6 Hz, 1H, Ar-H of naphthoquinone), 7.11 (s, 1H, Ar-H of naphthoquinone), 7.09 (d, *J* = 7.6 Hz, 1H, Ar-H of naphthoquinone), 7.00 (t, *J* = 7.5 Hz, 1H, Ar-H of phenyl group), 6.80–6.57 (m, 3H, CONH and 2Ar-H of phenyl group). 3.37–3.30 (m, 2H, NH-CH_2_), 3.30–3.21 (m, 2H, Ar-CH_2_), 3.00 (septet, *J* = 6.8 Hz, 1H, CH), 2.49–2.43 (m, 2H, CH_2_CO), 2.39 (s, 3H, Ar-CH_3_), 2.34–2.27 (m, 2H, CH_2_CO), 1.85–1.74 (m, 2H, CH_2_), 1.67–1.57 (m, 2H, CH_2_), 1.56–1.46 (m, 2H, CH_2_), 1.17 (d, *J* = 6.8 Hz, 6H, 2CH_3_). ^13^C- NMR (100 MHz, CDCl3) *δ* 182.59, 181.24, 172.62, 171.96, 146.84, 144.80, 140.90, 140.61, 140.49, 137.42, 135.17, 128.85, 128.12, 126.90, 125.29, 124.51, 119.21, 118.03, 39.06, 36.88, 35.79, 29.06, 28.08, 26.99, 26.22, 25.28, 21.47, 19.72. ESI-MS *m*/*z*: 490.2 [M + H]^+^. HRESI-MS calcd. for C_29_H_36_N_3_O_4_^+^ ([M + H]^+^) 490.2700, found 490.2706.

### 3.2. Biology

#### 3.2.1. IDO1 Enzymatic Assay

##### Materials

IDO-1 (CP produced, Lot. No. 20160706); NLG-919 (Selleckchem, Houston, TX, USA. Cat. No. S7111); Catalase (Sigma, St. Louis, MO, USA. Cat. No. C9322-5G); L-tryptophan (Sigma, St. Louis, MO, USA. Cat. No. 93659-10G); ascorbate (Sigma, St. Louis, MO, USA. Cat. No. 11140-250G); methylene blue (Sigma, St. Louis, MO, USA. Cat. No. M9140-100G); DMSO (Sigma, St. Louis, MO, USA. Cat. No. D2650); 96-well plate (Corning Inc., Corning, NY, USA. Cat. No. 3635).

##### Experimental Methods

(1) Prepare Compound

(1) Prepare 100× compound of the final desired highest inhibitor concentration in reaction by 100% DMSO. For example, if compounds test at 10 μM, then prepare 1 mM of compound solution in this step. Then dilute compound with DMSO in 3-fold mode to obtain different concentrations. Mark as source plate. (2) Add 100 μL of 100% DMSO to column 1 and column 12 for background control and no compound high control respectively.

(2) Prepare Assay Plate

Transfer 2 μL of each well from the 96-well source plate to a 96-well assay plate.

(3) Enzyme Reaction
  1. Prepare 2× enzyme solution. Add IDO-1 in assay buffer.
  2. Prepare 2× substrate solution. Add L-tryptophan, ascorbate, methylene blue, catalase in the assay buffer.
  3. Assay plate already contains 2 μL of compound in 100% DMSO.
  4. Transfer 2× enzyme solution to the assay plate. Add 100 μL of 2× enzyme solution to each well of the 96-well assay plate.
  5. Incubate at room temperature for 10 min.
  Transfer 2 ×
  1. × substrate solution to each well of the 96-well assay plate.
  2. Read OD_321_ kinetically by Spectramax (Molecular Devices, LLC, San Jose, CA, USA).
(4) Curve Fitting
  1. Copy data from Spectramax program. Percent inhibition = (max-conversion)/(max − min) × 100. “max” stands for high control; “min” stands for low control.
  2. Fit the data in XL-Fit to obtain IC_50_ values. Equation used is: Y = Bottom + (Top − Bottom)/(1 + 10^((LogIC_5_0 − X) × Hill Slope)

#### 3.2.2. HDAC Enzymatic Assay

##### Materials

HDAC1 (BPS Bioscience Inc., San Diego, CA, USA. Cat. No. 50051); DMSO (Sigma, St. Louis, MO, USA. Cat. No. D2650); 84-well plate (Perkin Elmer Inc., Waltham, MA, USA. Cat. No. 6007279)

##### Experimental Methods

(1) Prepare 1× assay buffer (modified Tris Buffer).
(2) Compound serial dilution: Transfer compounds to assay plate by Echo in 100% DMSO. The final fraction of DMSO is 1%.
(3) Prepare enzyme solution in 1× assay buffer.
(4) Add trypsin and Ac-peptide substrate in 1× assay buffer to make the substrate solution.
(5) Transfer 15 μL of enzyme solution to assay plate or for low control transfer 15 μL of 1× assay buffer.
(6) Incubate at room temperature for 15 min.
(7) Add 10 μL of substrate solution to each well to start reaction.
(8) Incubate at room temperature for 60 min.
(9) Read the plate on Synergy MX with excitation at 355 nm and emission at 460 nm.
(10) Curve fitting: Fit the data in Excel to obtain inhibition values using Equation (1).

Inh% = (Max − Signal)/(Max − Min) × 100(1)

Fit the data in XL-Fit to obtain IC_50_ values using Equation (2);

Y = Bottom + (Top − Bottom)/(1 + 10^((LogIC_50_ − X) × Hill Slope)) (2)

Y is %inhibition and X is compound concentration.

#### 3.2.3. Anti-Proliferation Assay in A549 Cell Line

Cell viability was measured by the CellTiter-Glo Luminescent (CTG) assay. A549 cells (ATCC, Cat. No.CCL-185) were seeded (2000 cells/well) in 96-well plates and cells were left to incubate at 37 °C for 24 h. After 24 h, prepare compounds with 5-fold dilution in DMSO to the compound plate (200-fold of the final concentration) and transfer 5 μL from the compound plate to 95 μL intermediate medium in the intermediate plate (10-fold of the final concentration). Then transfer 10 μL from the intermediate plate to the cell plates. The final DMSO is 0.5%. After continuing to incubate for 48 h, add 50 μL CTG reagent to each well and read luminescence according to CTG product manual.

#### 3.2.4. Anti-Proliferation Assay in Primary Endothelial Cells (ECs)

Primary endothelial cells (ECs) were isolated from male rats and cultured in DMEM supplemented with 10% FBS, 100 units/mL penicillin-streptomycin at 37 °C in a humidified atmosphere under 5% CO_2_. ECs were plated in a 96-well plate at a density of 2 × 104 cells/well. After serum starved overnight, ECs were exposed to a gradient concentration of 200 μM for 24 h. Cell viability was evaluated by Cell Counting Kit-8 (CCK-8) assay. The results were expressed as fold changes by normalizing the data to the control values.

### 3.3. Molecular Docking

Protein structures were reconstructed by SWISS-MODEL homology-modelling server [25] using IDO1 and HDAC1 as templates. Then the AutoDock 4.2 software with the Lamarckian genetic algorithm was used for the molecular docking to predict the binding modes. Crystal structure of human IDO1 in complex (PDB code: 5EK2) and HDAC1 (PDB code: 4BKX) were retrieved from Protein Data Bank (www.rcsb.org).

The ADT and grid calculations were carried out using grid box dimensions of 60 × 60 × 60 Å with 0.375 Å spacing followed by the space creations around the binding site of IDO1 protein. Pretreatment of the compounds and the IDO receptor structure for docking was carried out with the AutoDockTools program suite (mgltools.scripps.edu). Default parameters were used and one hundred independent docking runs were conducted for each compound.

The docking study of HDAC1 and **33d** was carried out using the same method as described above.

### 3.4. QM/MM Simulation

After docking, best poses of **33d**-protein complexes with dominant conformation underwent QM/MM simulations by GROMACS + MOPAC + RM1 packages as mentioned by Gonçalves and colleagues [26], and the procedure adopted to this task is described in Supporting Information. System of simulation was set up using charmm27 forcefield and box of TIP3P water molecules. The selection and characterization of the quantum atoms were performed by the visualization of the docking system with the PyMOL 2.2 software. Regarding IDO1, select ligand, heme and the neighboring residues (Tyr126, Phe164, Phe226, Arg231, Gly262 and Ala264) as the quantum atoms. Regarding HDAC1, select ligand, znic and the neighboring residues (His140, His141 and Tyr303) as the quantum atoms. The link atoms (LA) were set between the α and β carbon atoms of each amino acid cited above. These atoms constituted the QM region of the system, and the remaining atoms of the protein, the counter ions and the solvent molecules constituted the MM region. The condition for the QM/MM simulation was the frame of the classic simulation. All these steps were performed at 310 K and 1 bar of pressure and the integration step was 1 fs or half of the integration step used in the classic simulation, in order to make possible to deal with the bond vibrations between C and H, usually unnoticeable at 2 fs. After 20ns of QM/MM simulation, poses at the last frames in the simulations were selected for the analysis and visualization by PyMol software.

### 3.5. Molecular Dynamics Simulation

Molecular dynamics (MD) simulations of compound **33d** along with IDO1 and HDAC1, using the GROMACS 5.1.2 software [27] employed to track the motions of individual atoms. The ACPYPE server was used to generate the topology and force field parameters for ligand [28]. Each ligand-target complex was soaked in a dodecahedral box of TIP3P water molecules with a margin of at least 10 Å from the complex surface. Sodium and chloride counter ions were added to preserve electroneutrality. Each complex was subjected to energy minimization with 50,000 steps of steepest descent minimization. Afterwards, two MD equilibration phases were simulated. Firstly, a 500-ps step under the NVT ensemble was applied, using an integration time step of 2.0 fs at a temperature of 310 K. Another 500 ps NPT equilibration step was then executed at 1 bar pressure to equilibrate the system. The MD production phase was subsequently conducted using the NPT ensemble at 310 K and the V-rescale temperature coupling algorithm. The pressure in the system was adjusted to 1 atm under isotropic molecule-based scaling using the Parrinello-Rahman pressure coupling method. Long-range electrostatics was treated with the particle mesh Ewald (PME) algorithm. Finally, molecular dynamics simulation was performed at a timescale of 100 ns and in 50,000,000 steps. The MD trajectories were analyzed by plotting the root mean square deviation (RMSD) for each frame against time, as well as the root mean square fluctuation (RMSF) of the protein backbone, and the hydrogenbonding interactions between the target and the ligand were also recorded. The g_mmpbsa procedure was applied to analyze the binding energy during the last 30 ns of the MD simulation. Data were visualized using GraphPad Prism 7 (GraphPad Software Inc., San Diego, CA, USA).

## 4. Conclusions

A kind of *ortho*-quinone compounds from natural product were firstly reported to have better IDO1 inhibition activity than *para-*quinones. To discover the IDO1 and HDAC dual inhibitors for cancer treatment by taking advantages of immunotherapeutic and epigenetic drugs, saprorthoquinone was chosen as a hit compound through a pharmacophore fusion strategy. A more effective IDO1 and HDAC dual inhibitor **33d** was obtained, which showed balanced activity against both IDO1 (IC_50_ = 0.73 μM) and HDAC1 (IC_50_ = 0.46 μM). Importantly, structure-activity relationship studies revealed that an *ortho*-quinone pharmacophore group was necessary for the IDO inhibition and a *N*-(2-aminophenyl) amide pharmacophore group was necessary for the HDAC inhibition, which combined by an appropriate five carbons linker. Moreover, the binding modes of these inhibitors to the enzyme active site were studied by molecular docking, showed that the coordination bonding interaction and hydrogen bonding interaction were the major interaction between potent inhibitors and IDO1. The hydrogen bond with Gly262 and Leu234 of IDO1 appeared to confer increased potency to this class of inhibitors which explained the higher activity of both **19** and **33d**. This study provides a new strategy for future design of IDO1/HDAC dual inhibitors as antitumor agents. Structural optimization of lead compound **33d** remains to be further investigated in our lab.

## Figures and Tables

**Table 1:** Structures and IDO1 inhibitory activities of saprorthoquinone and its analogues.

Compds.	Structure	IDO1 Inhibition @ 10 μM (%)	Compds.	Structure	IDO1 Inhibition @ 10 μM (%)
**1**	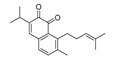	85%	**10**	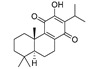	2%
**4**	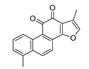	65%	**11**	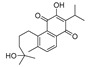	2%
**5**	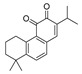	81%	**12**	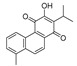	13%
**6**	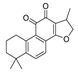	66%	**13**	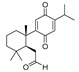	6%
**7**	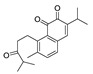	86%	**14**	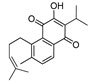	30%
**8**	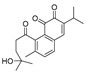	73%	**15**	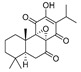	3%
**9**	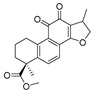	61%	**16**	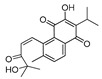	3%
Control	Epacadostat	100%			

**Table 2:** IDO1 inhibitory and antiproliferative activities of the target compounds.

Compound	IDO1 IC_50_ (μM) ^a^ or % Inhibition at 10 μM	A549 IC_50_ (μM)
**1**	1.76 ± 0.27 (85%)	9.97
**17**	0.74 ± 0.15 (90%)	12.84
**18**	0.99 ± 0.24 (86%)	>20
**19**	0.51 ± 0.04 (78%)	14.48
**21a**	1.33 ± 0.01 (80%)	13.07
**21b**	64%	14.83
**21c**	64%	7.49
**21d**	65%	9.31
**21e**	66%	>20
**21f**	79%	>20
**21g**	74%	12.17
**24a**	0.72 ± 0.13 (91%)	11.82
**24b**	87%	15.54
**24c**	86%	11.05
NLG-919	0.10 ± 0.02	NT ^b^

^a^ IC_50_ values are the mean of at least two independent assays, presented as mean ± SD. ^b^ NT means not tested.

**Table 3:** HDAC1, IDO1 inhibitory and antiproliferative activities of the target compounds.

Compd.	IDO1 IC_50_ (μM) ^a^ or % Inhibition at 10 μM	HDAC1 IC_50_ (μM) ^a^	A549 IC_50_ (μM)
**27a**	0.72 ± 0.10 (88%)	>100	17.75
**27b**	84%	>100	9.51
**27c**	56%	>100	>20
**33a**	89%	>100	>20
**33b**	0.58 ± 0.11 (80%)	34.87 ± 0.75	19.06
**33c**	0.55 ± 0.02 (90%)	39.48 ± 0.74	>20
**33d**	0.71 ± 0.03 (82%)	0.46 ± 0.07	>20
NLG-919	0.10 ± 0.02 (85%)	NT ^b^	NT ^b^
SAHA	NT ^b^	0.015 ± 0.00012	NT ^b^

^a^ IC_50_ values are the mean of at least two independent assays, presented as mean ± SD. ^b^ NT means not tested.

## Supplementary Materials

The following are available online. Supporting information including the spectra of ^1^H, ^13^C-NMR, compilation of GROMACS + MOPAC + RM1 and general procedure of input file set-up in QM/MM Simulation, are available online.
