# Supplementary material for: Discovery of Novel Indoleamine 2,3-Dioxygenase 1 (IDO1) and Histone Deacetylase 1 (HDAC1) Dual Inhibitors Derived from the Natural Product Saprorthoquinone
Source: Molecules. 2020 Sep 30;25(19):4494. doi: 10.3390/molecules25194494 (PMC7582476; doi:10.3390/molecules25194494)
Supplement: Supplementary file 1 [file molecules-25-04494-s001.pdf]

Supporting Information

Discovery of Novel Indoleamine 2,3-Dioxygenase 1 (IDO1) and Histone Deacetylase 1 (HDAC1) Dual Inhibitors Derived from the Natural Product Saprorthoquinone

Table of Contents

1 Copies of ^{1}H NMR and ^{13}C NMR Spectrum for All Compounds S2

2 Software and General Procedure of QM/MM Simulation S20

1. Copies of ^{1}H NMR and ^{13}C NMR Spectrum for All Compounds

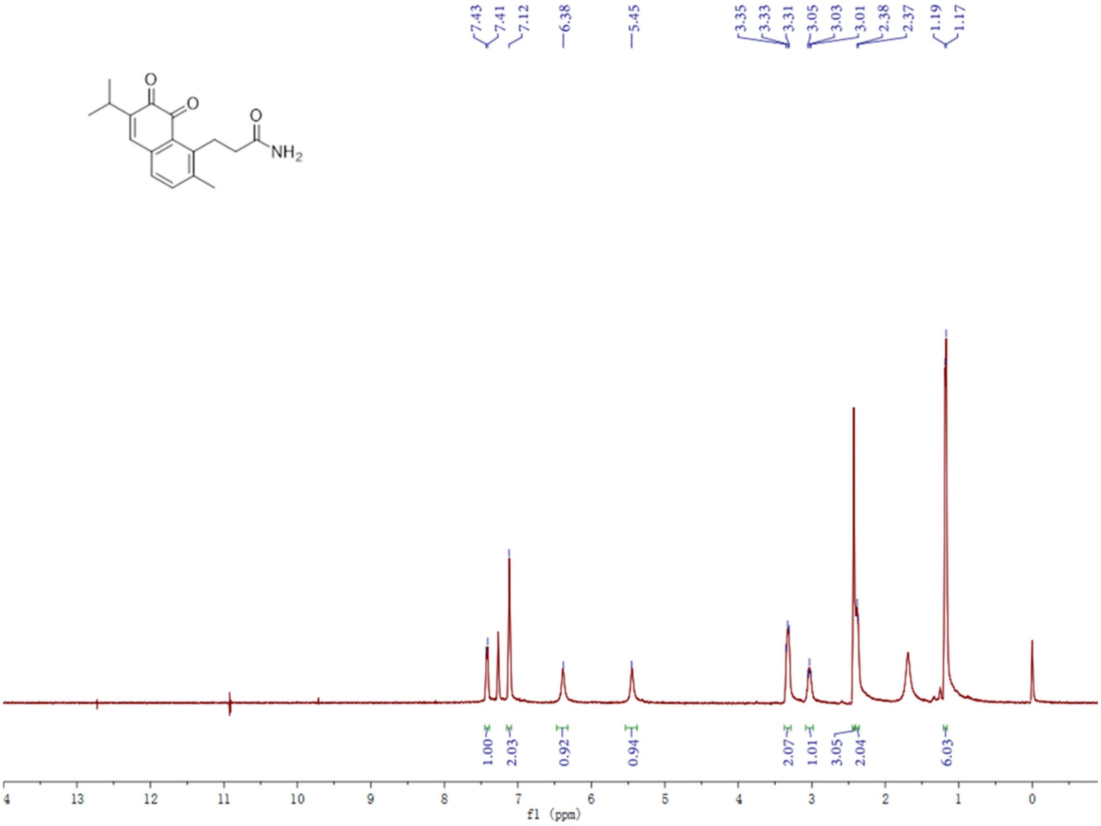

^{1}H NMR spectrum of compound **19**

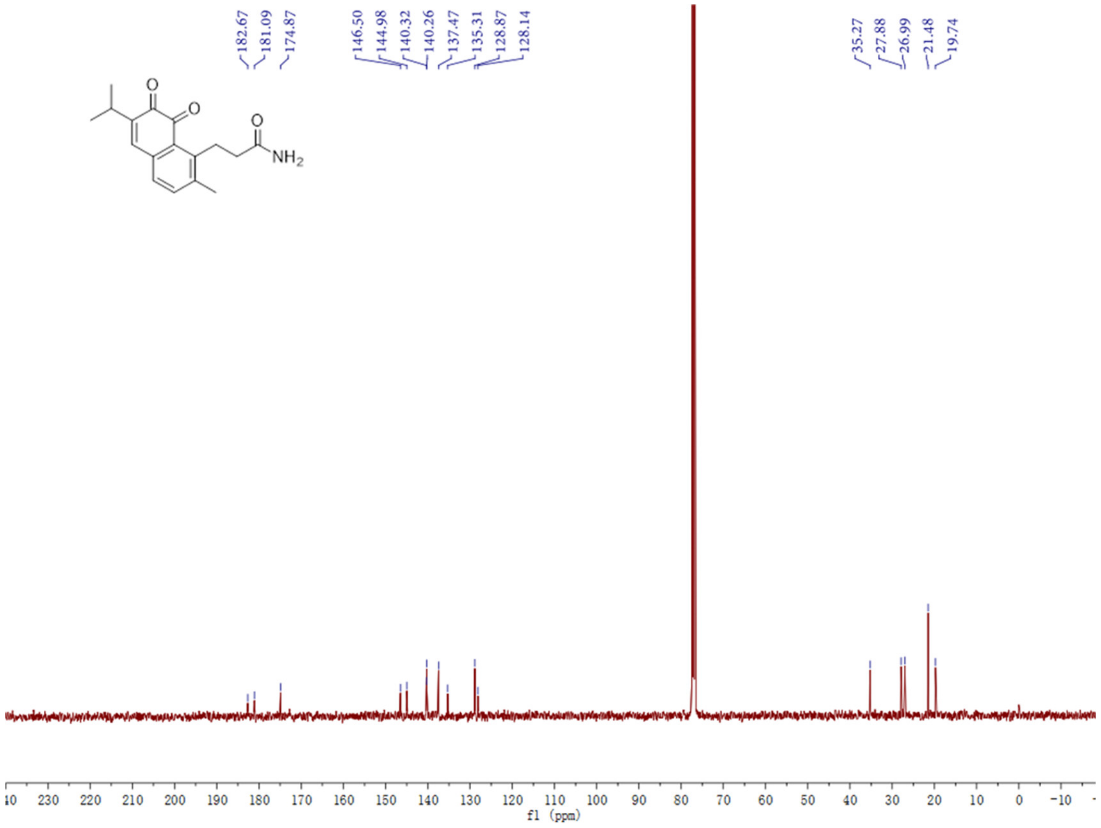

^{13}C NMR spectrum of compound **19**

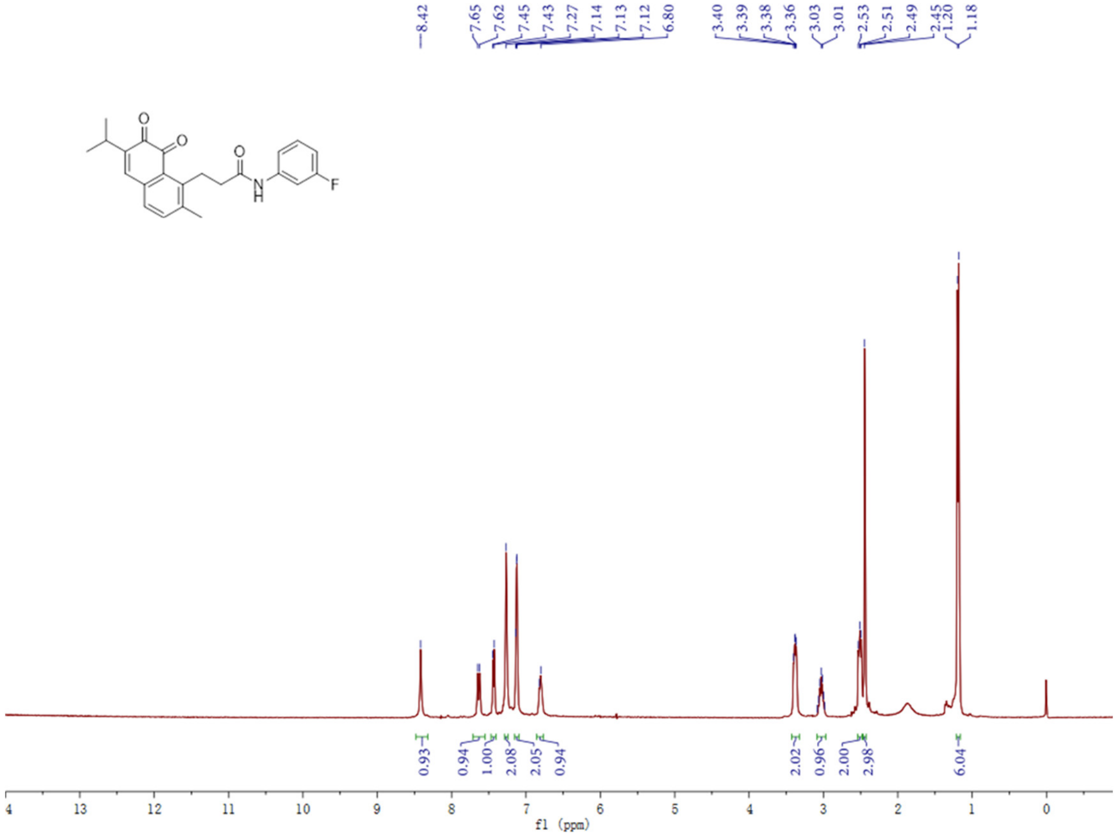

^{1}H NMR spectrum of compound **21a**

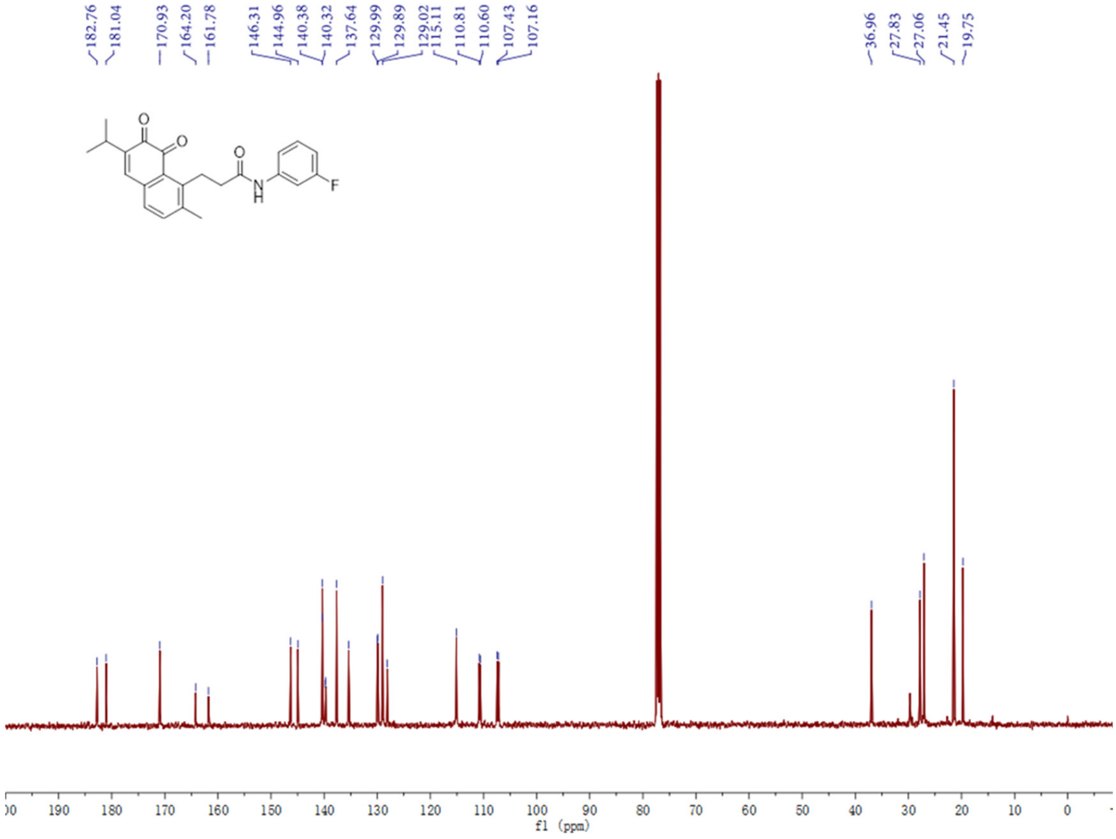

^{13}C NMR spectrum of compound **21a**

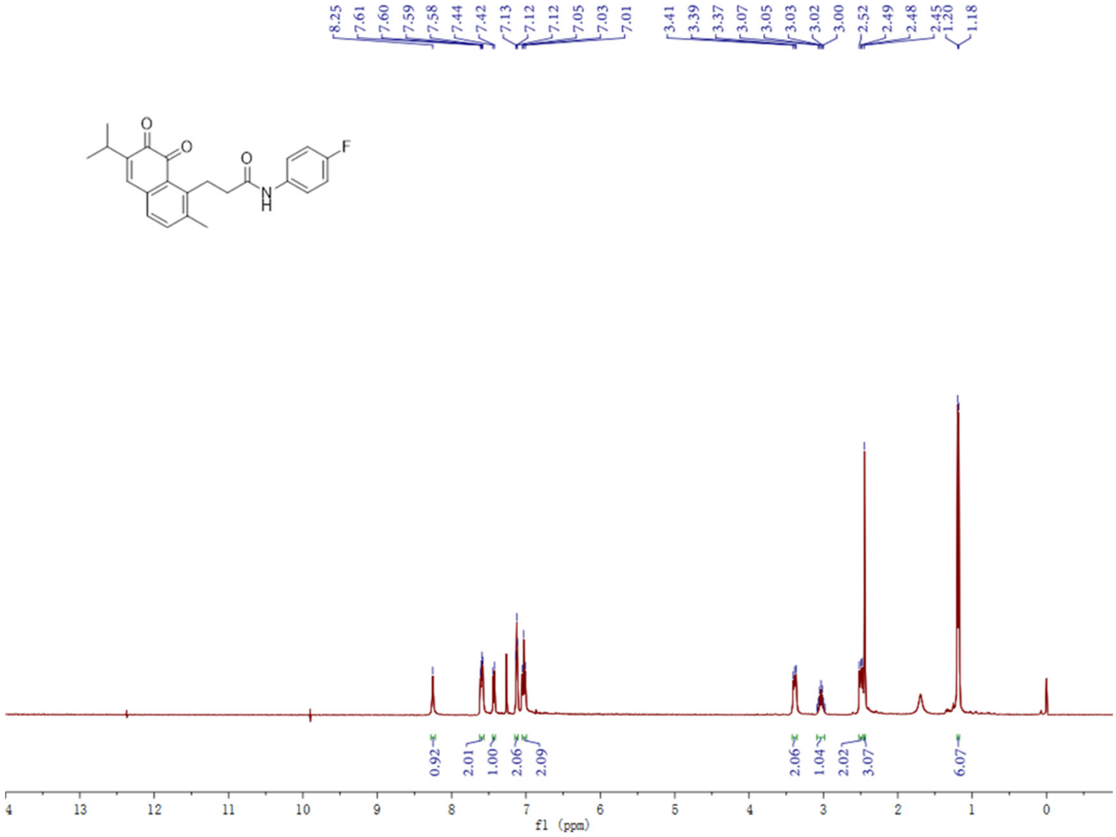

^{1}H NMR spectrum of compound **21b**

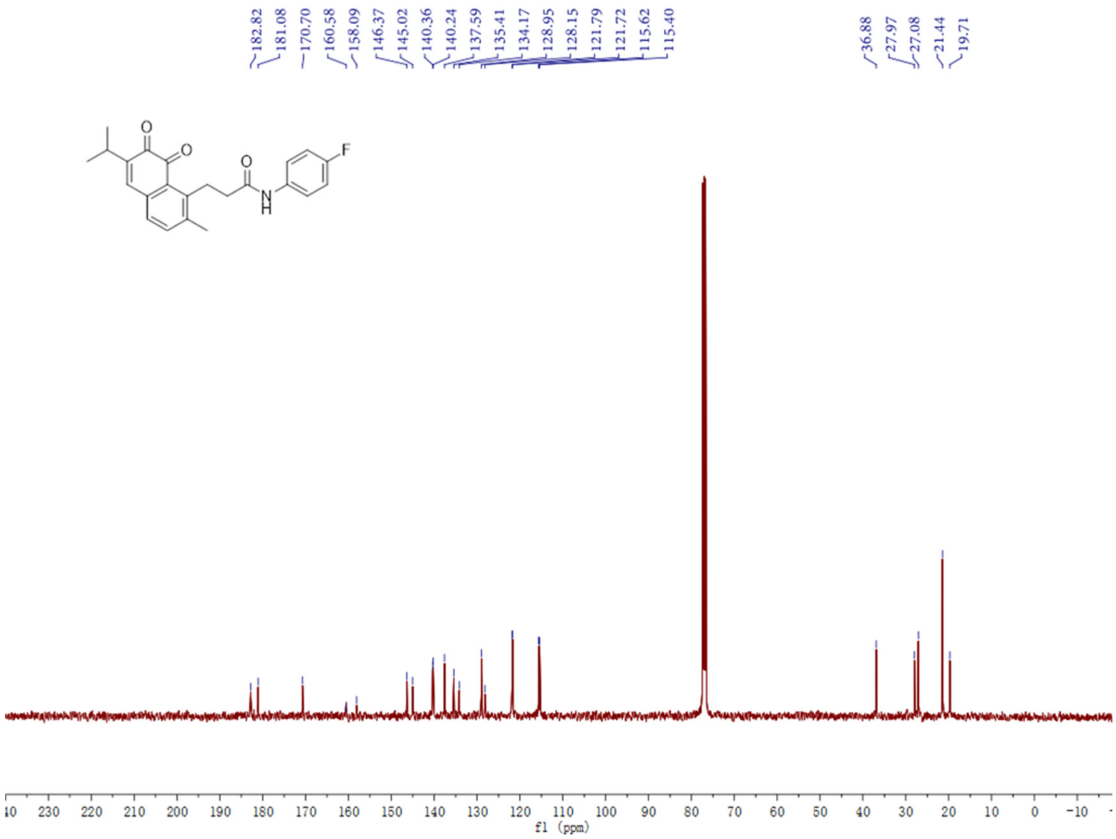

^{13}C NMR spectrum of compound **21b**

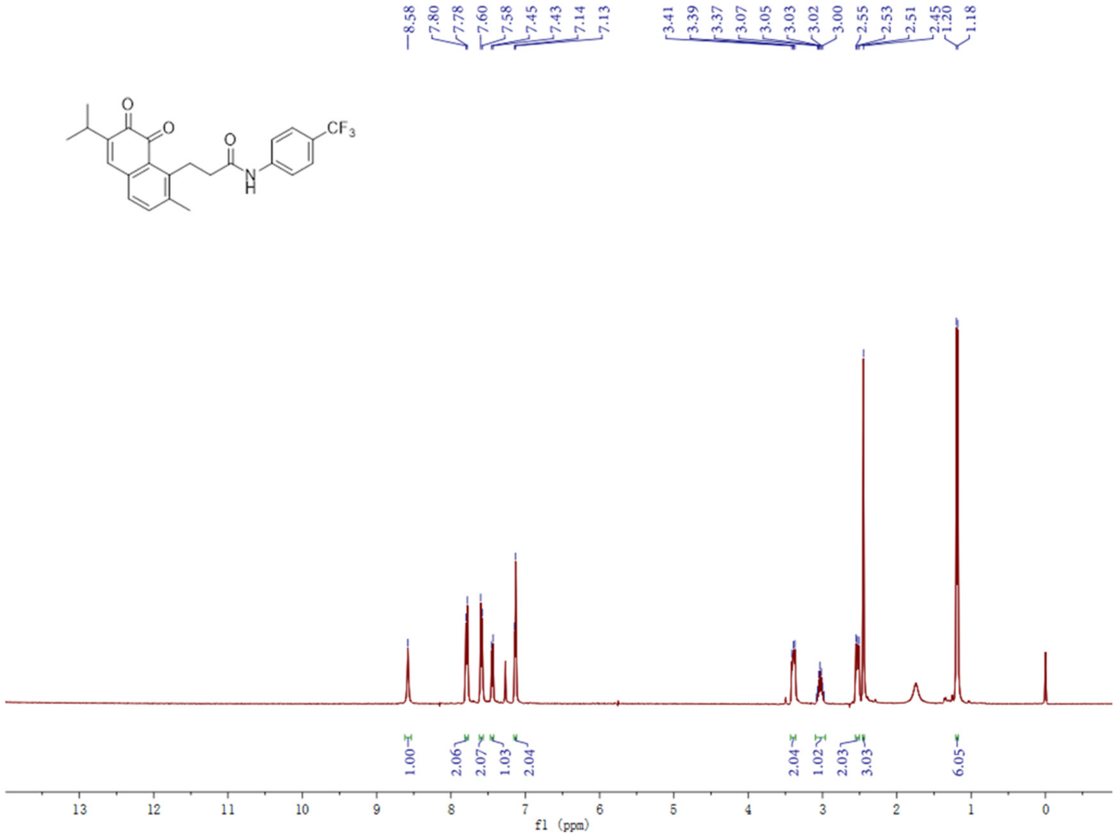

^{1}H NMR spectrum of compound **21c**

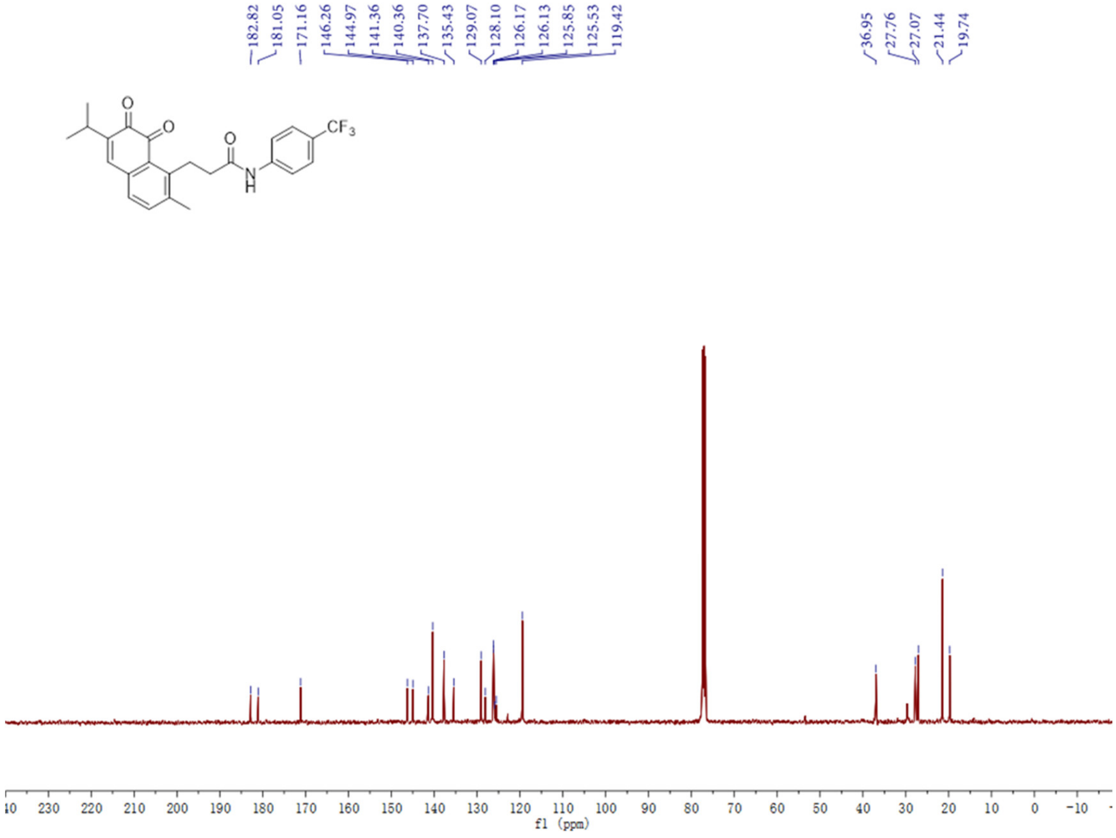

^{13}C NMR spectrum of compound **21c**

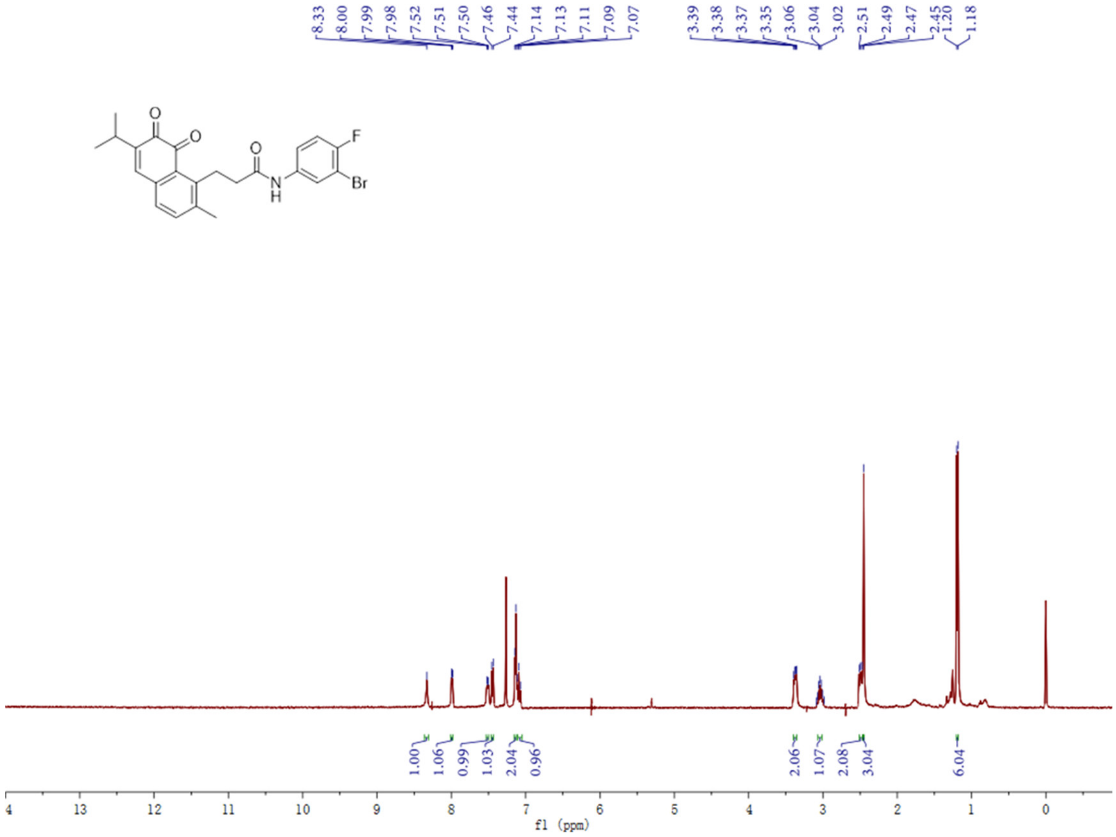

^{1}H NMR spectrum of compound **21d**

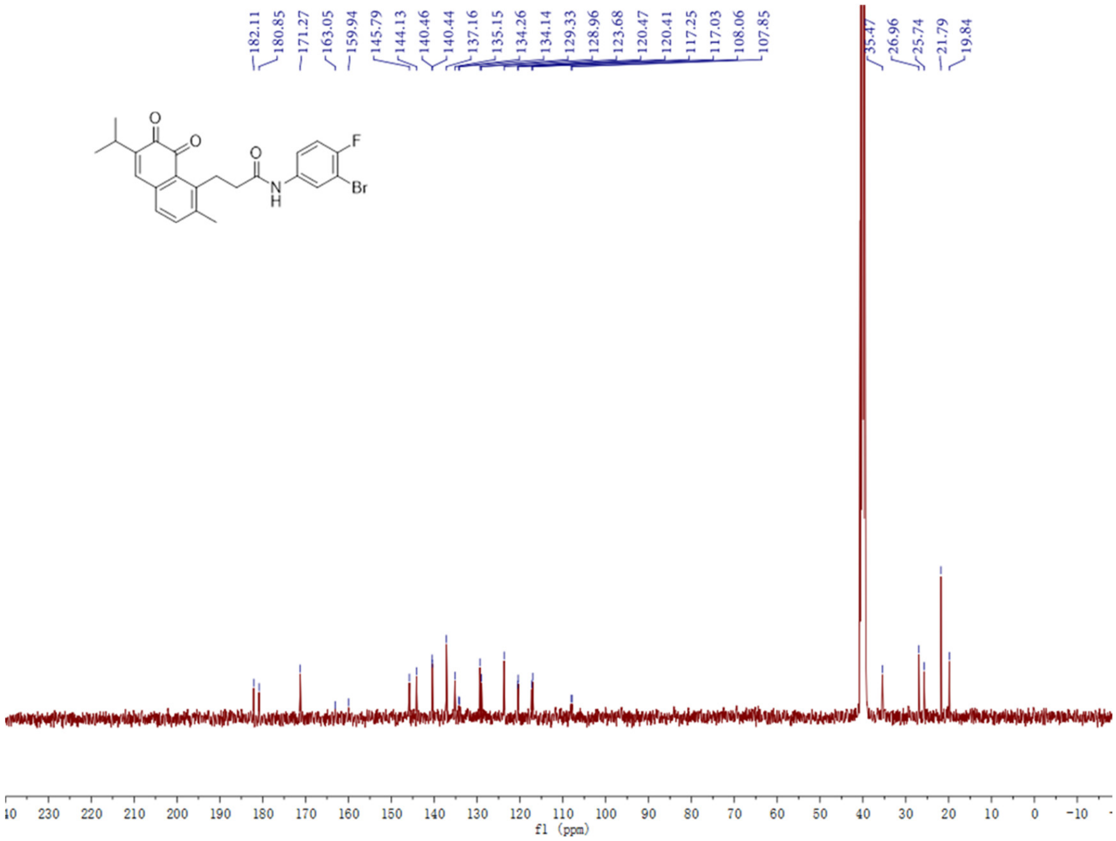

^{13}C NMR spectrum of compound **21d**

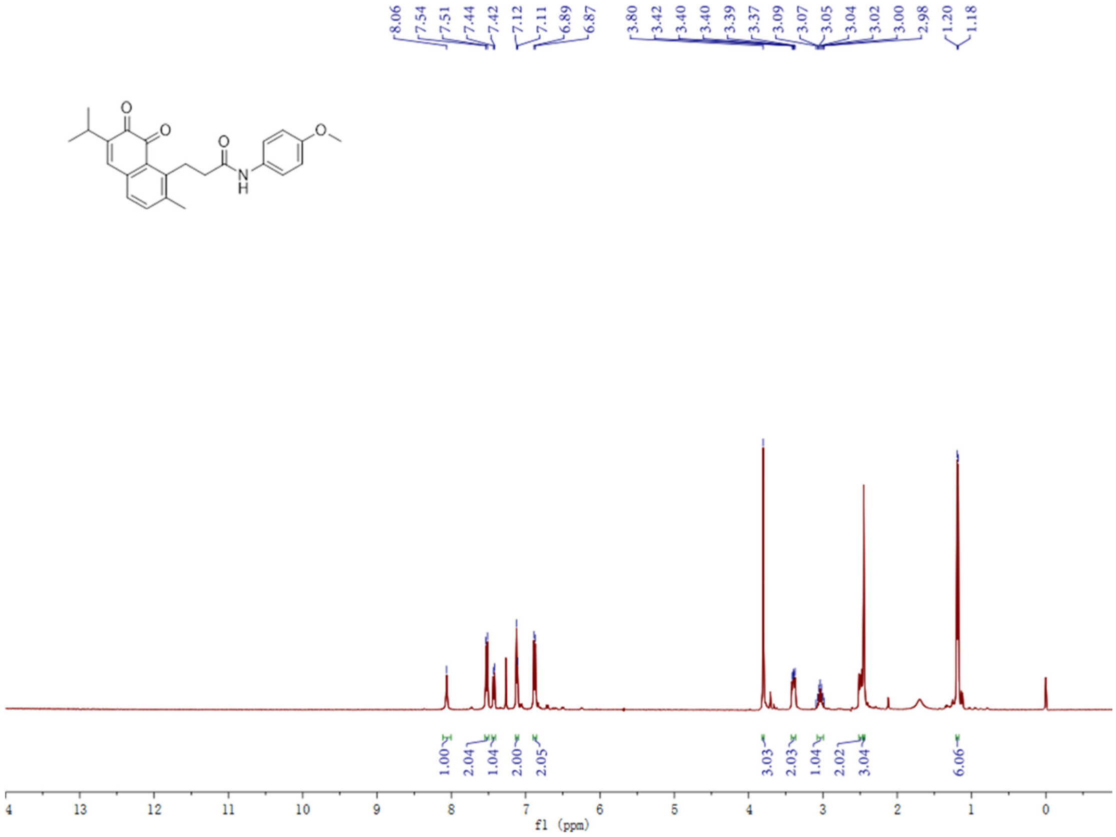

^{1}H NMR spectrum of compound **21e**

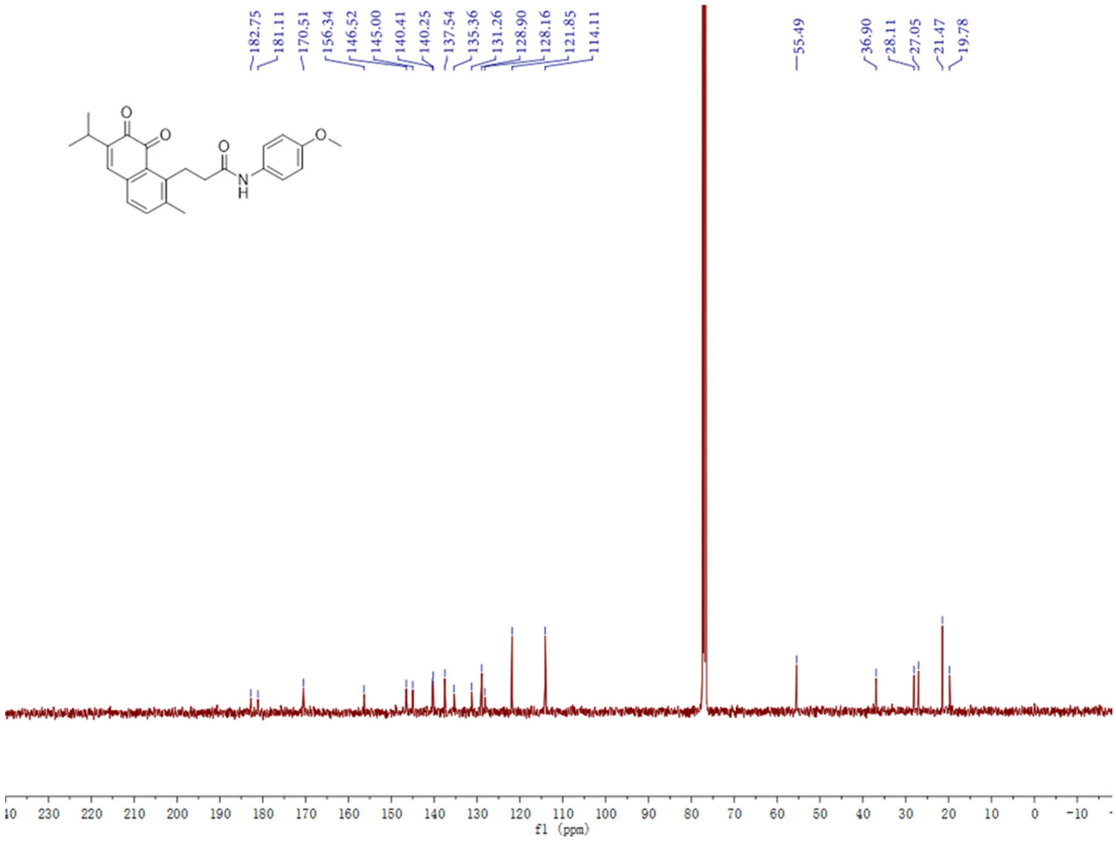

^{13}C NMR spectrum of compound **21e**

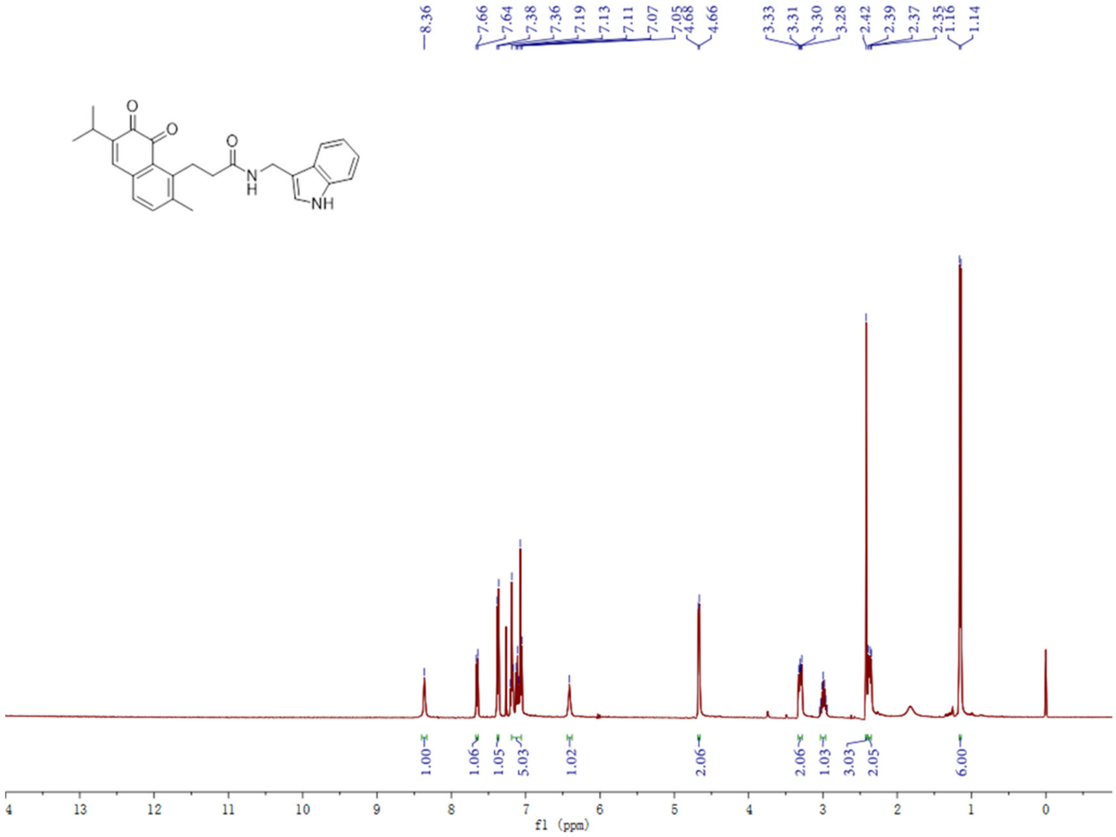

^{1}H NMR spectrum of compound **21f**

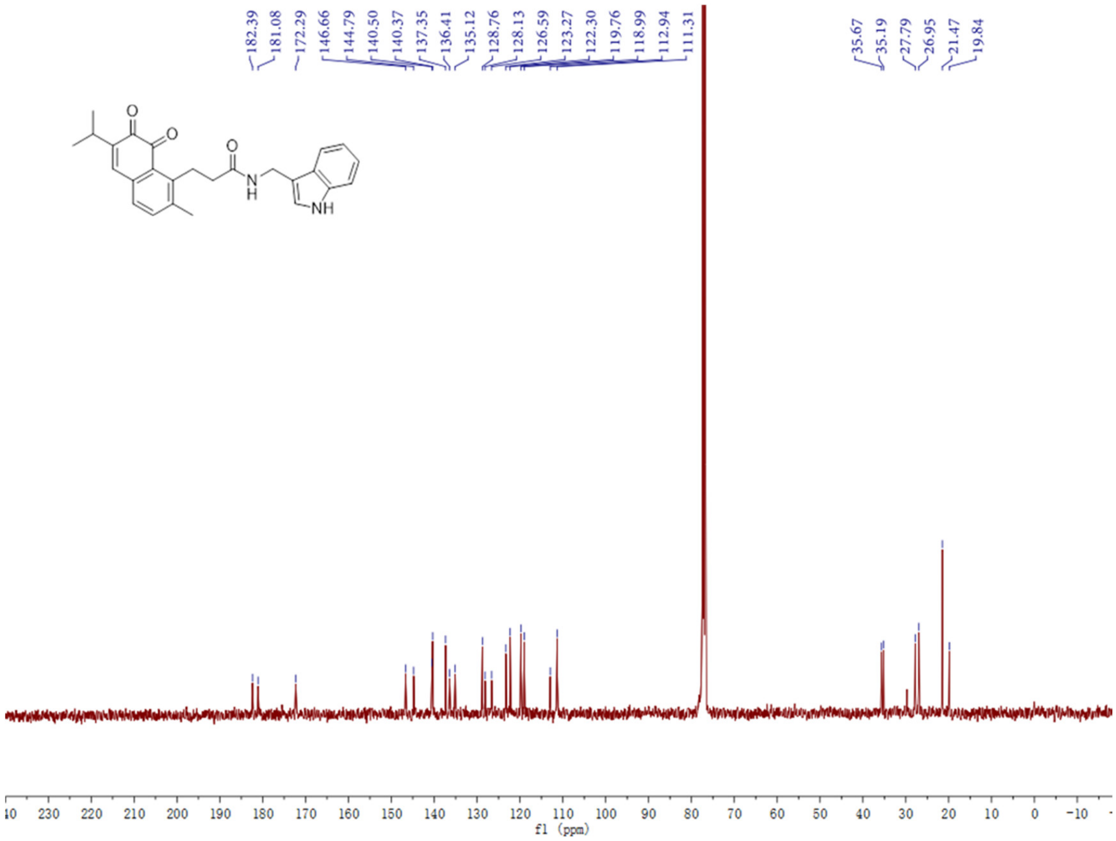

^{13}C NMR spectrum of compound **21f**

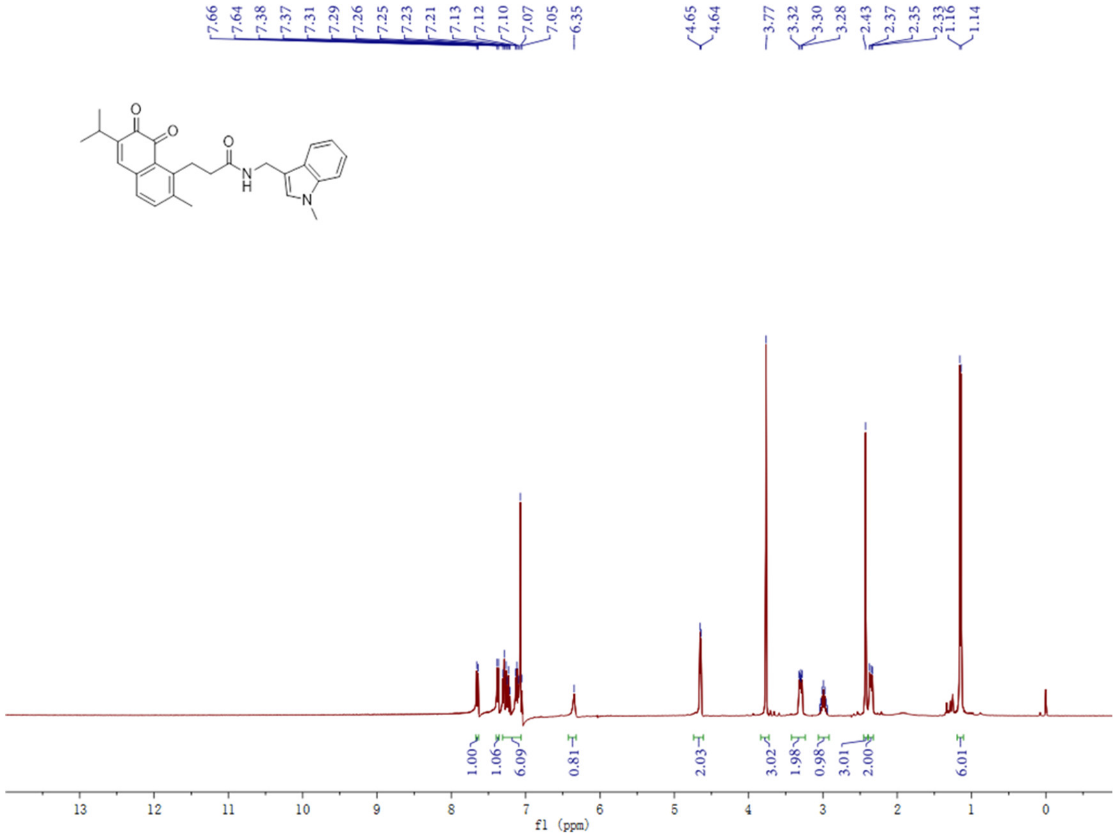

^{1}H NMR spectrum of compound **21g**

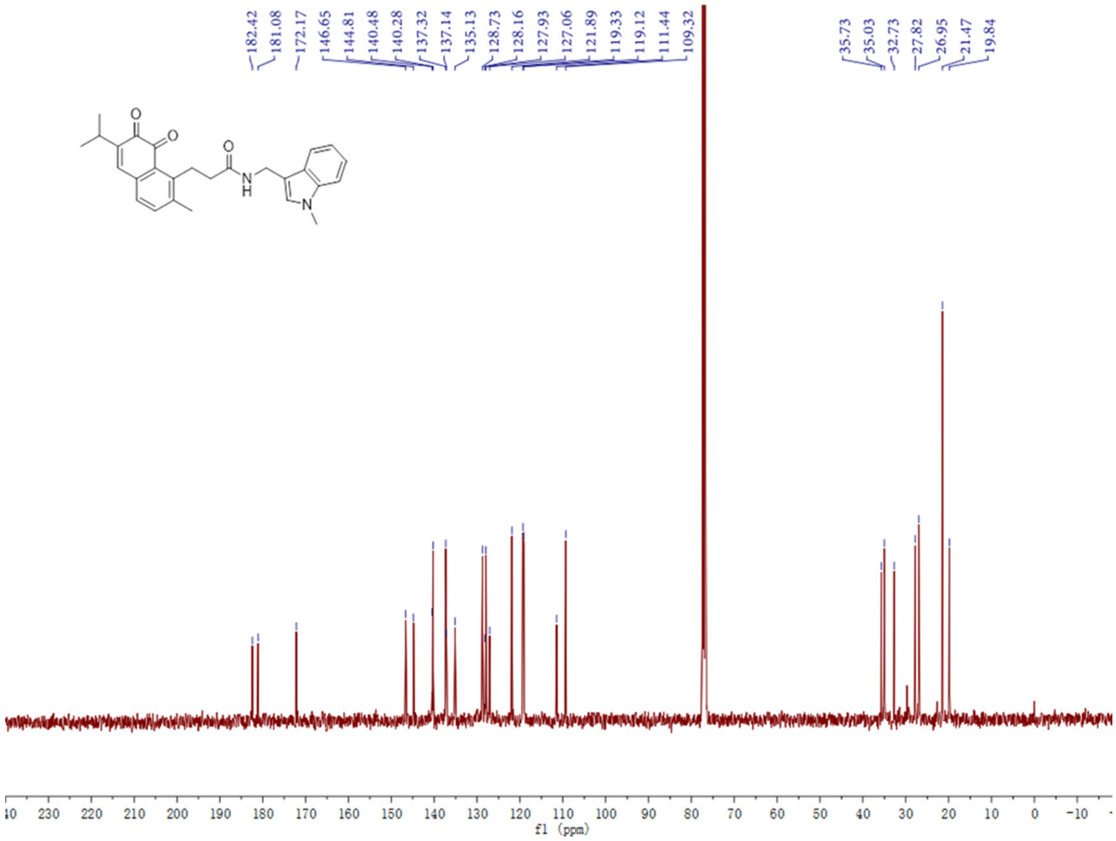

^{13}C NMR spectrum of compound **21g**

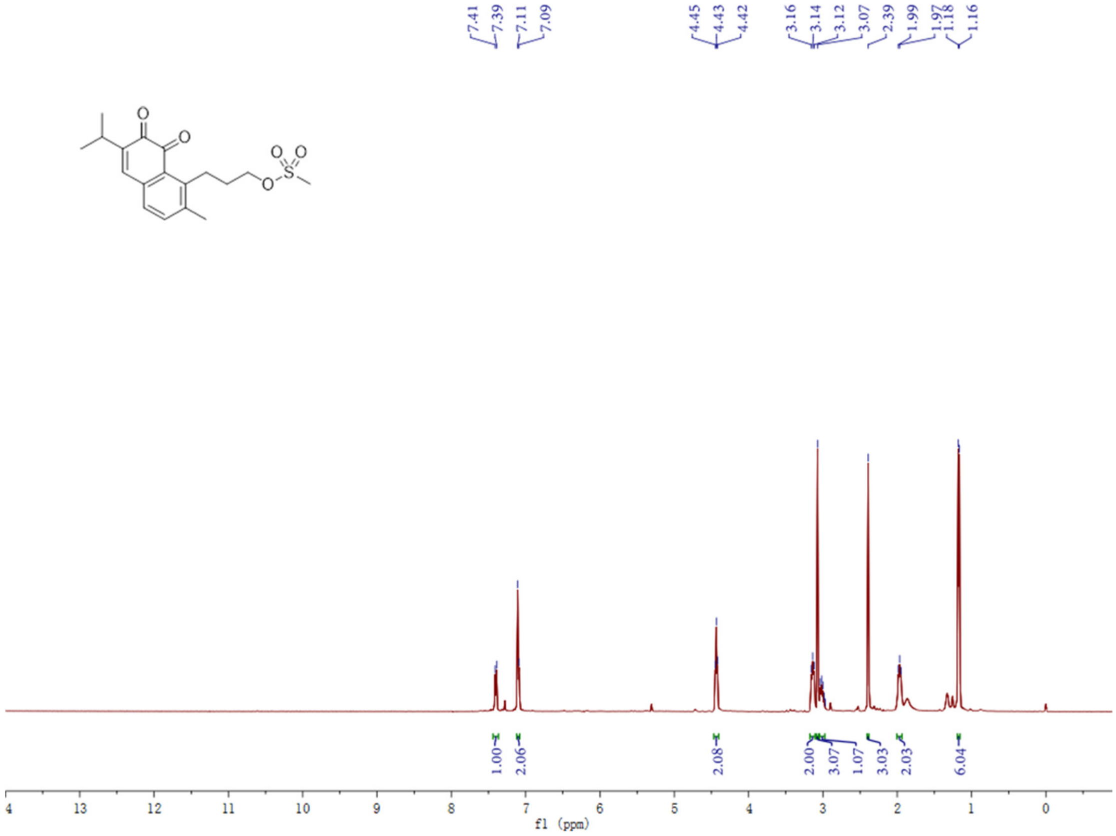

^{1}H NMR spectrum of compound **24a**

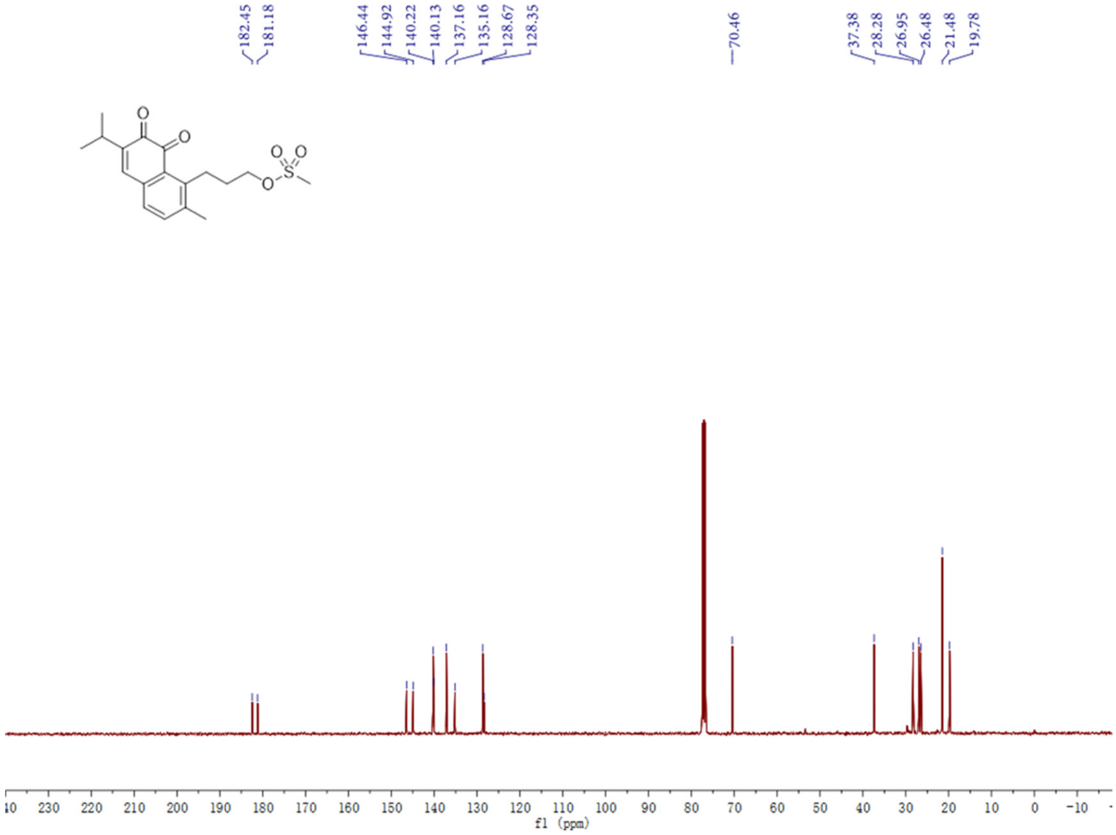

^{13}C NMR spectrum of compound **24a**

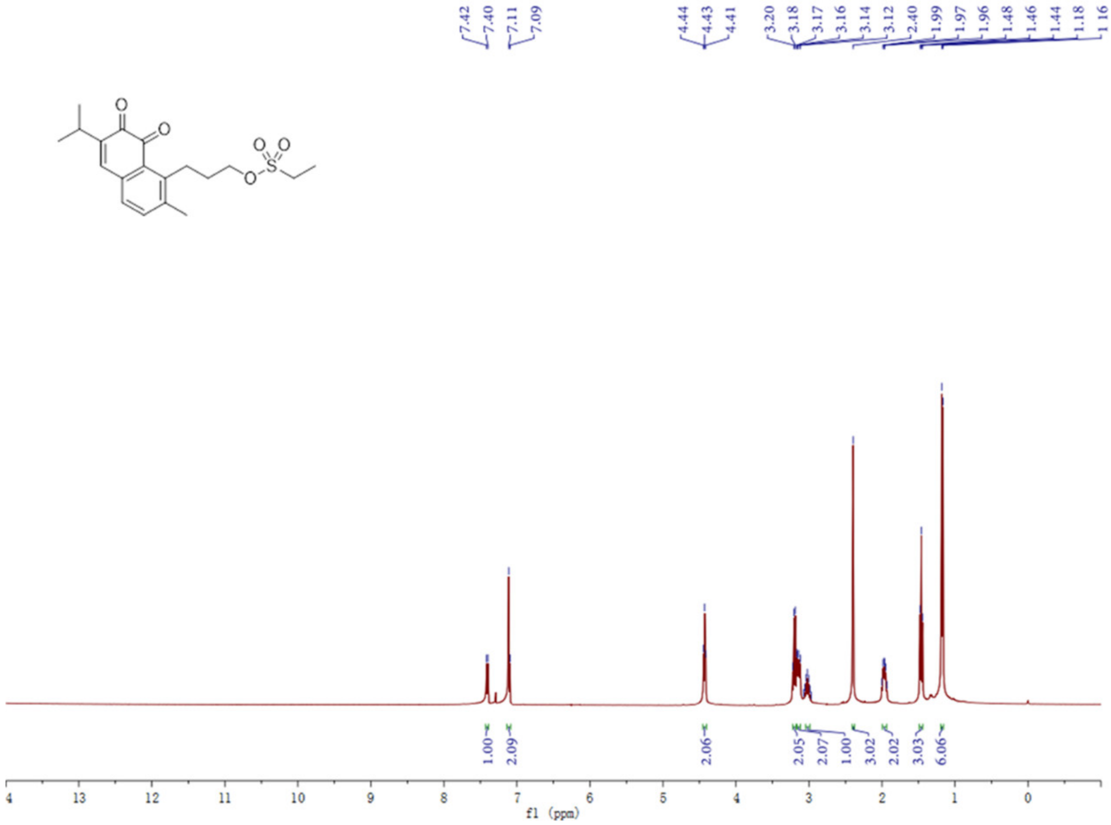

^{1}H NMR spectrum of compound **24b**

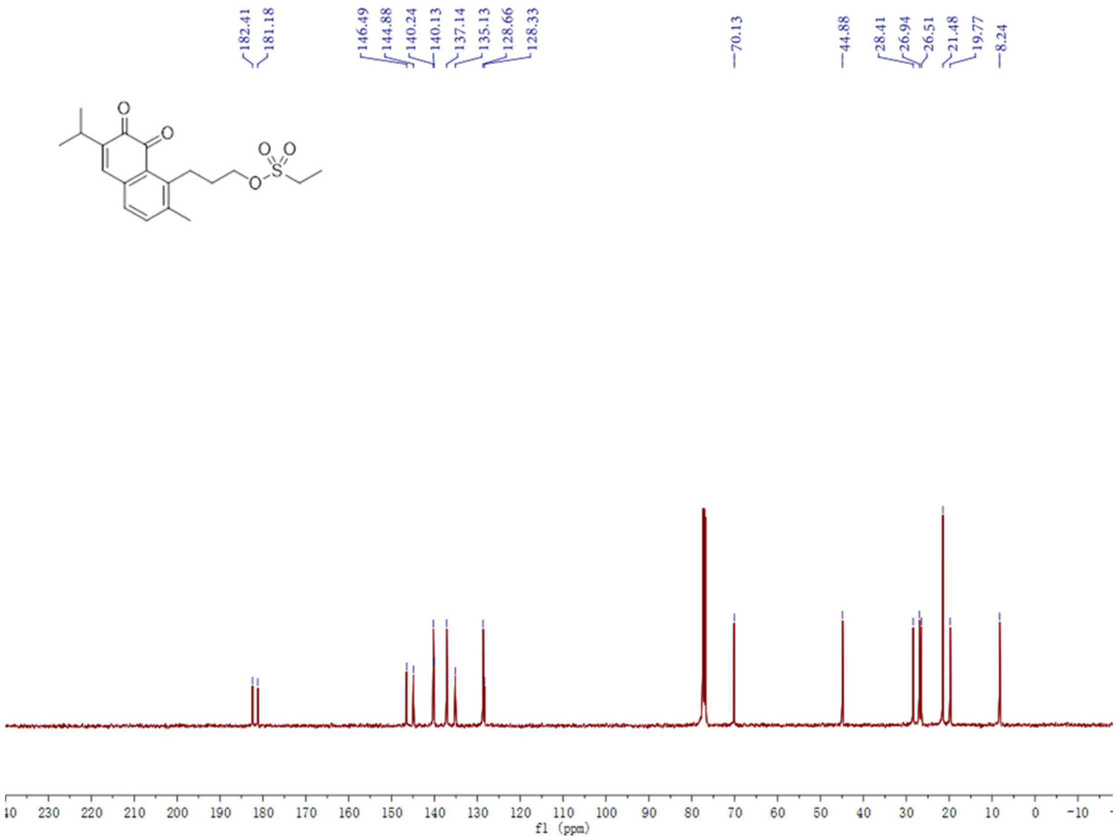

^{13}C NMR spectrum of compound **24b**

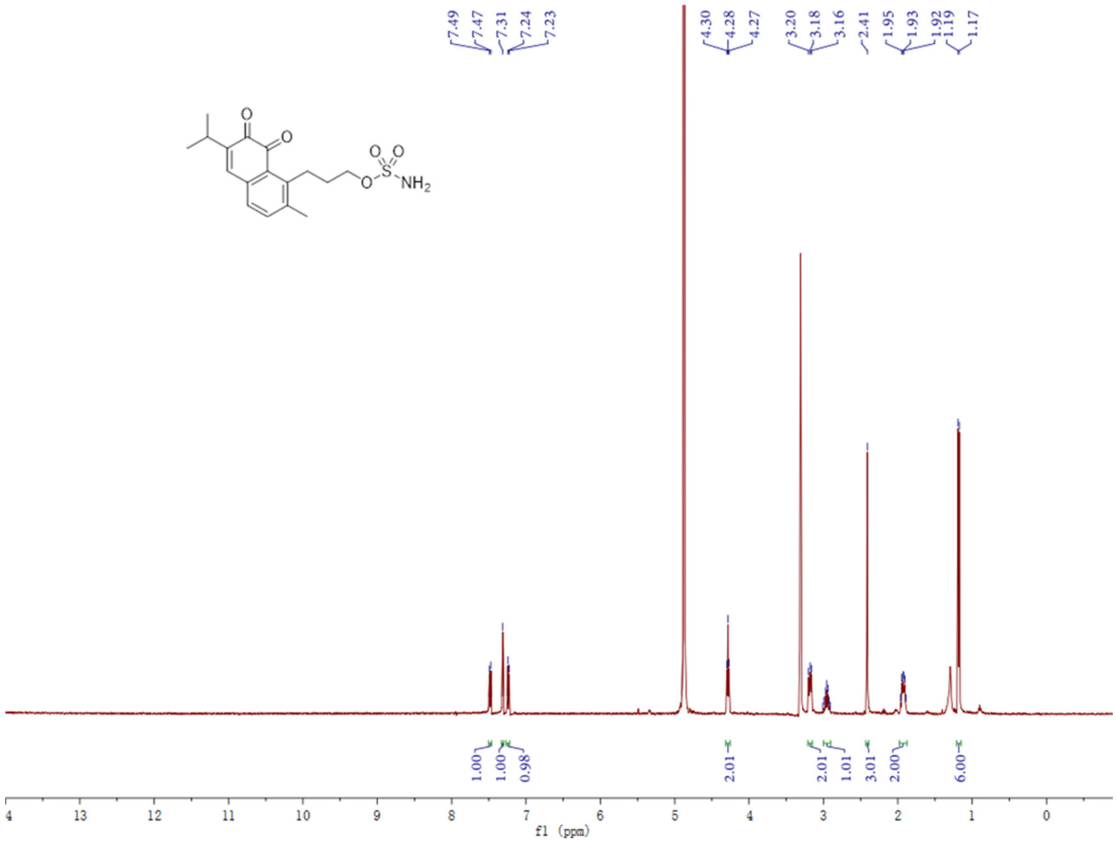

^{1}H NMR spectrum of compound **24c**

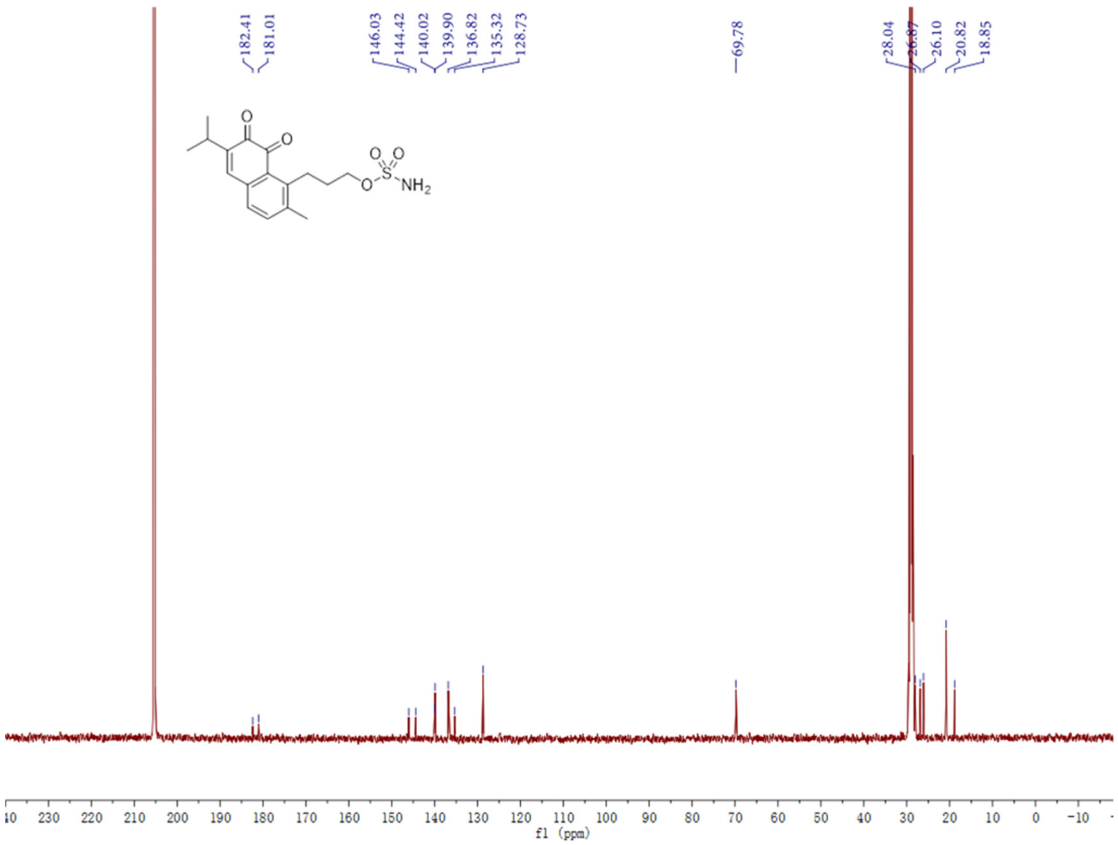

^{13}C NMR spectrum of compound **24c**

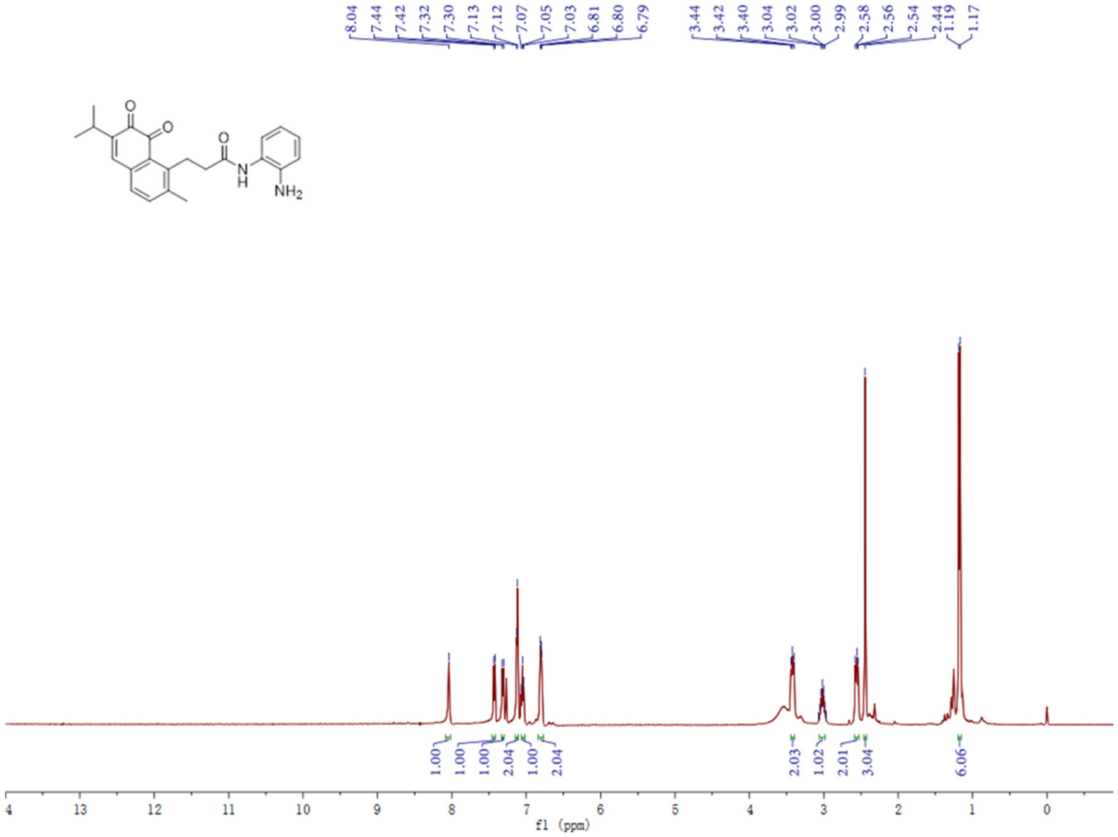

^{1}H NMR spectrum of compound **27a**

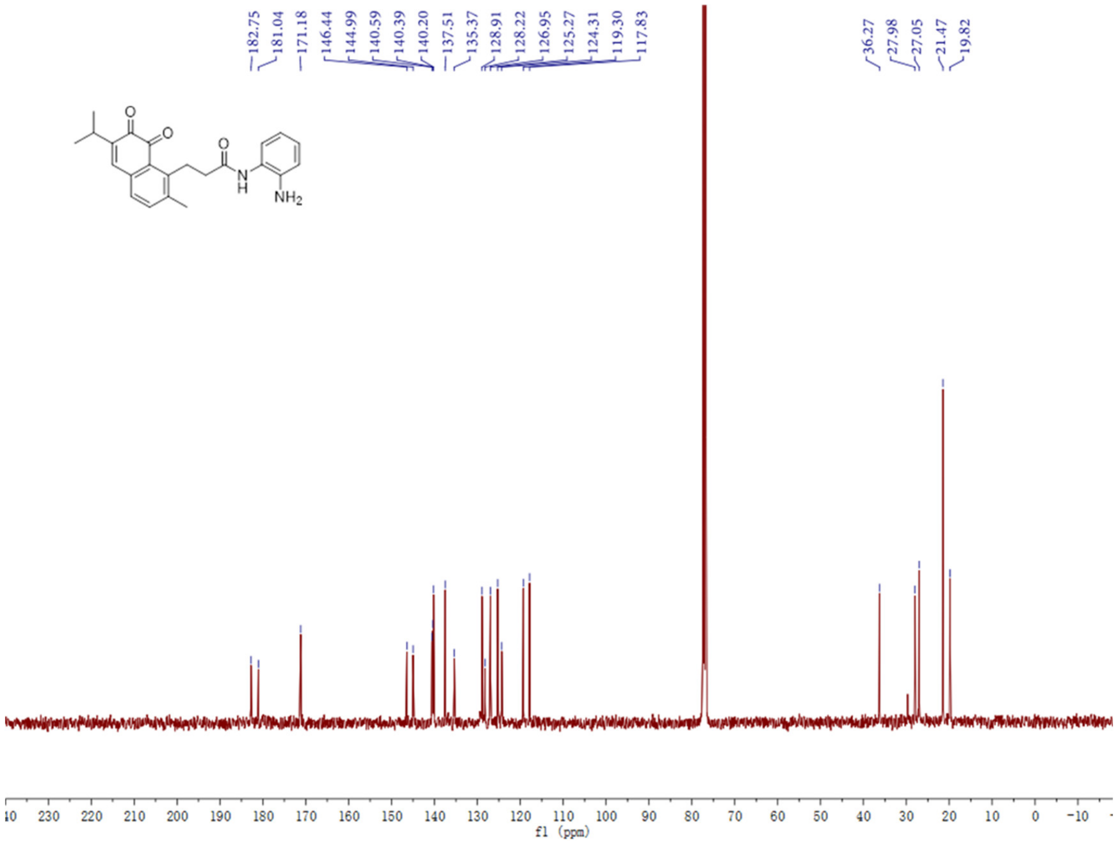

^{13}C NMR spectrum of compound **27a**

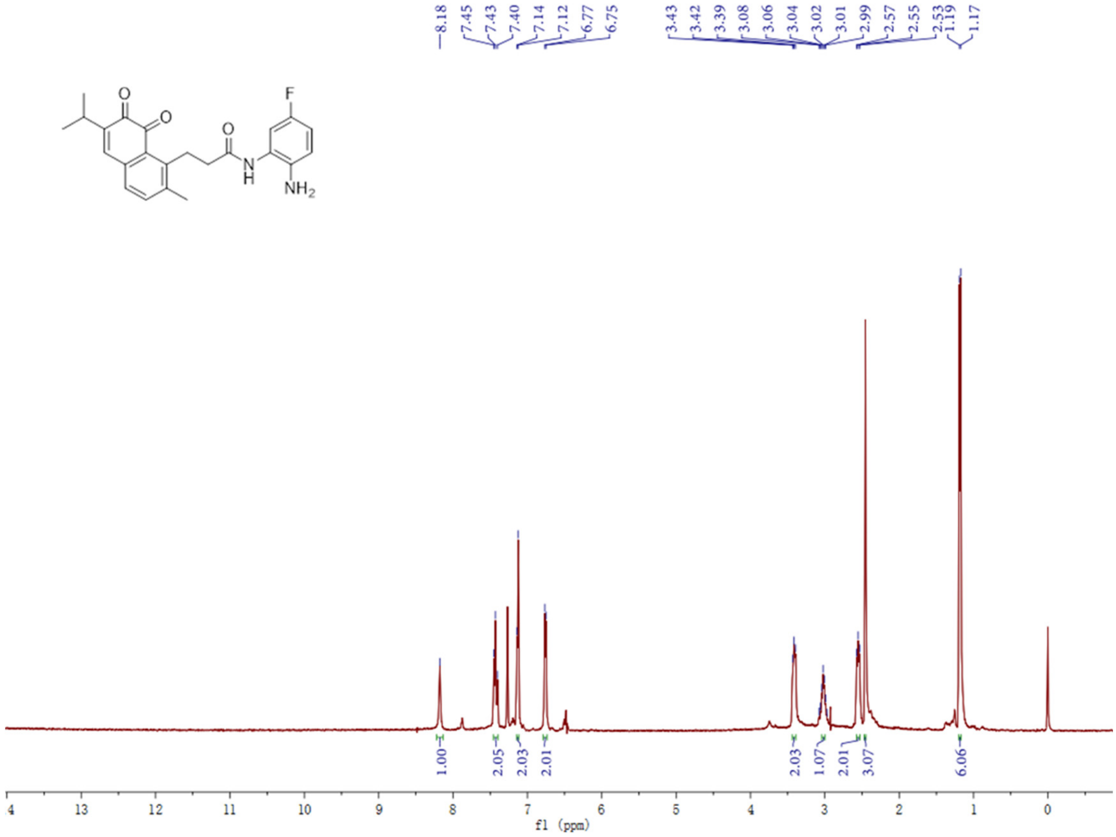

^{1}H NMR spectrum of compound **27b**

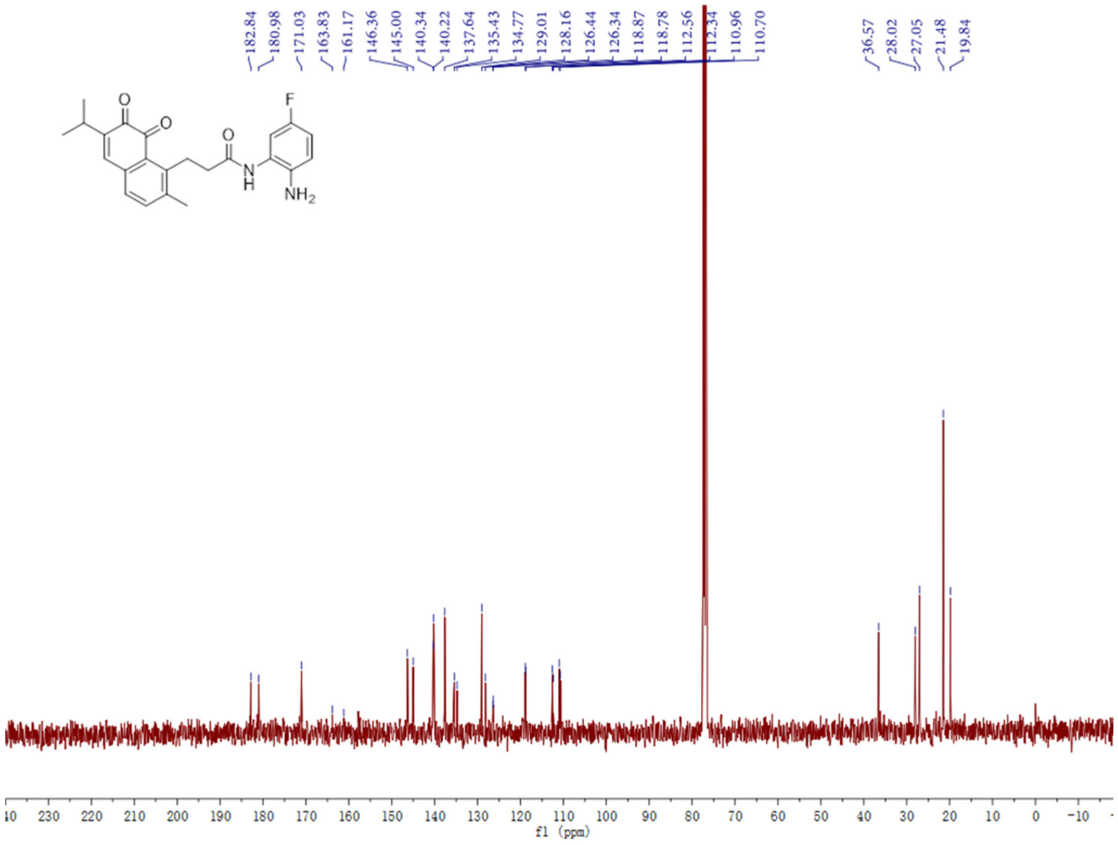

^{13}C NMR spectrum of compound **27b**

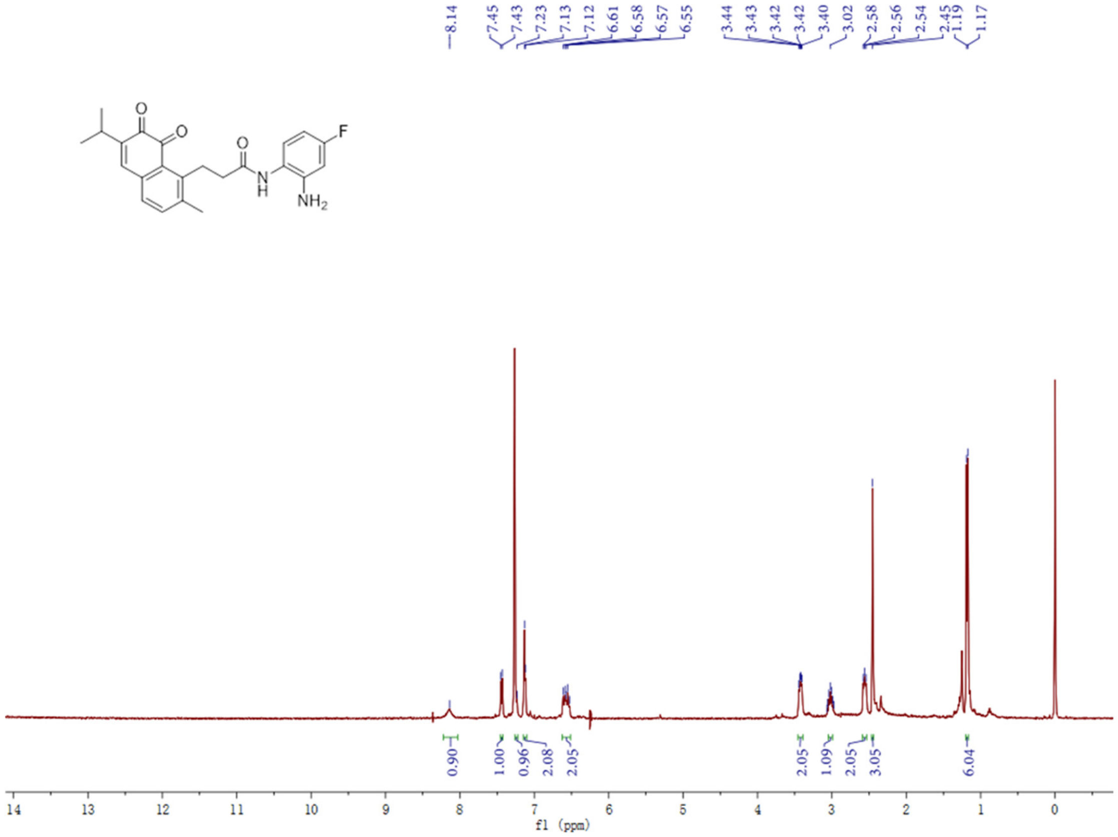

^{1}H NMR spectrum of compound **27c**

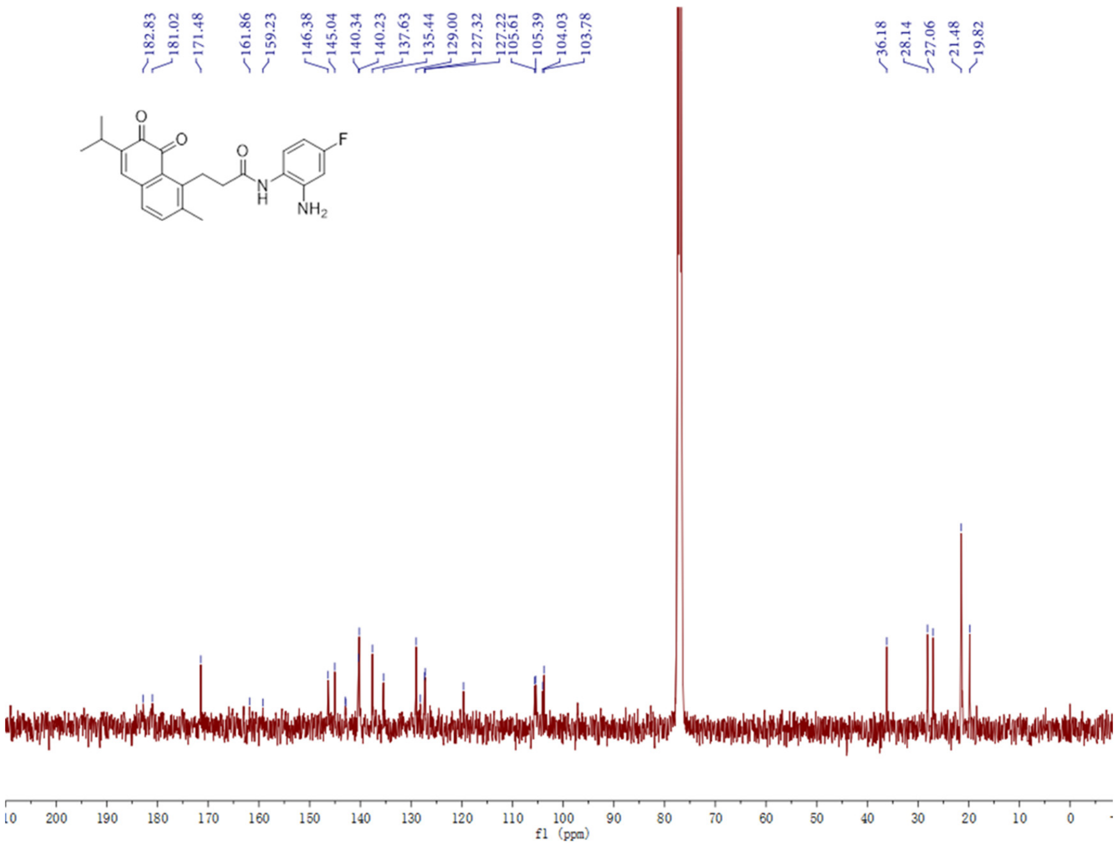

^{13}C NMR spectrum of compound **27c**

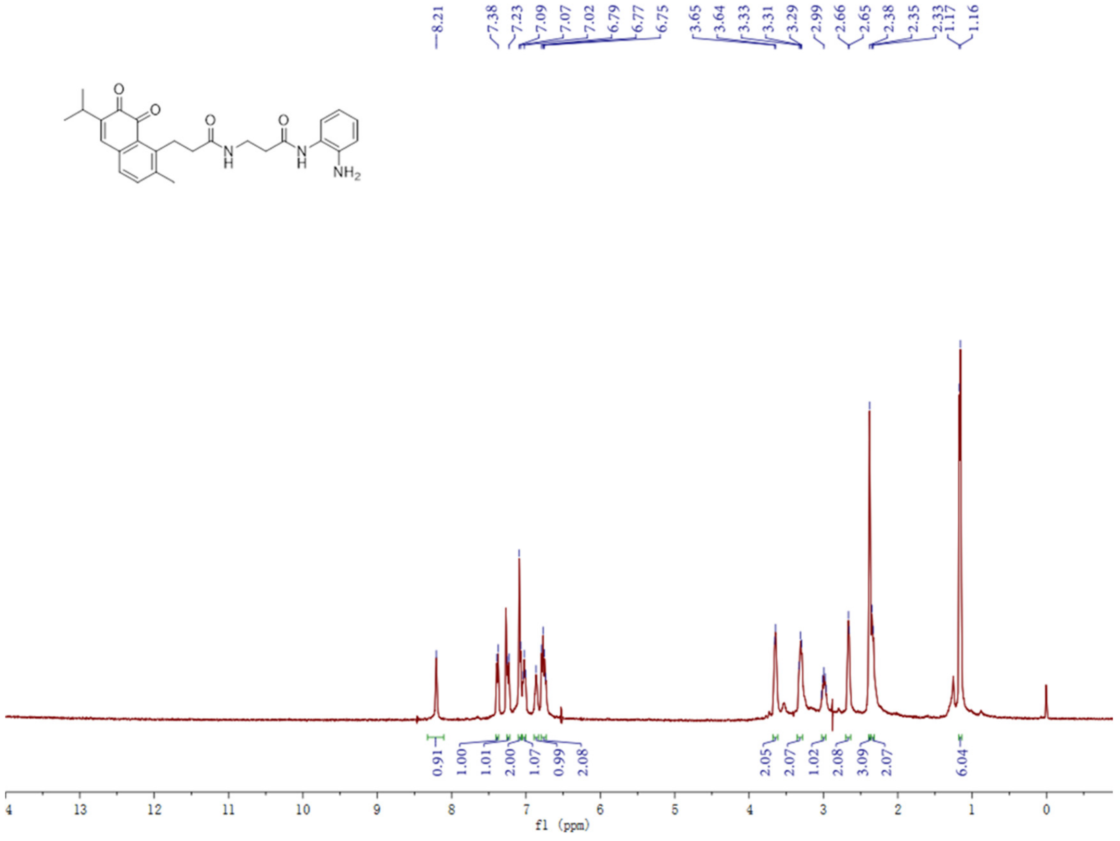

^{1}H NMR spectrum of compound **33a**

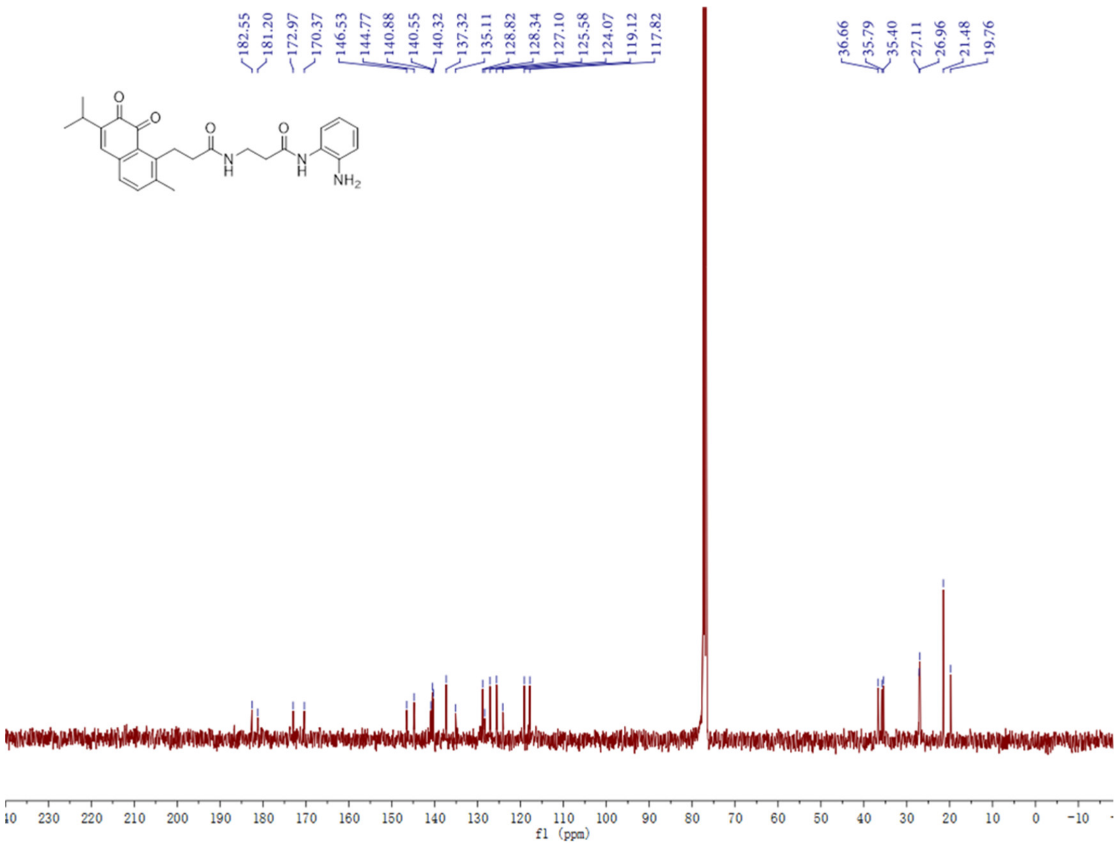

^{13}C NMR spectrum of compound **33a**

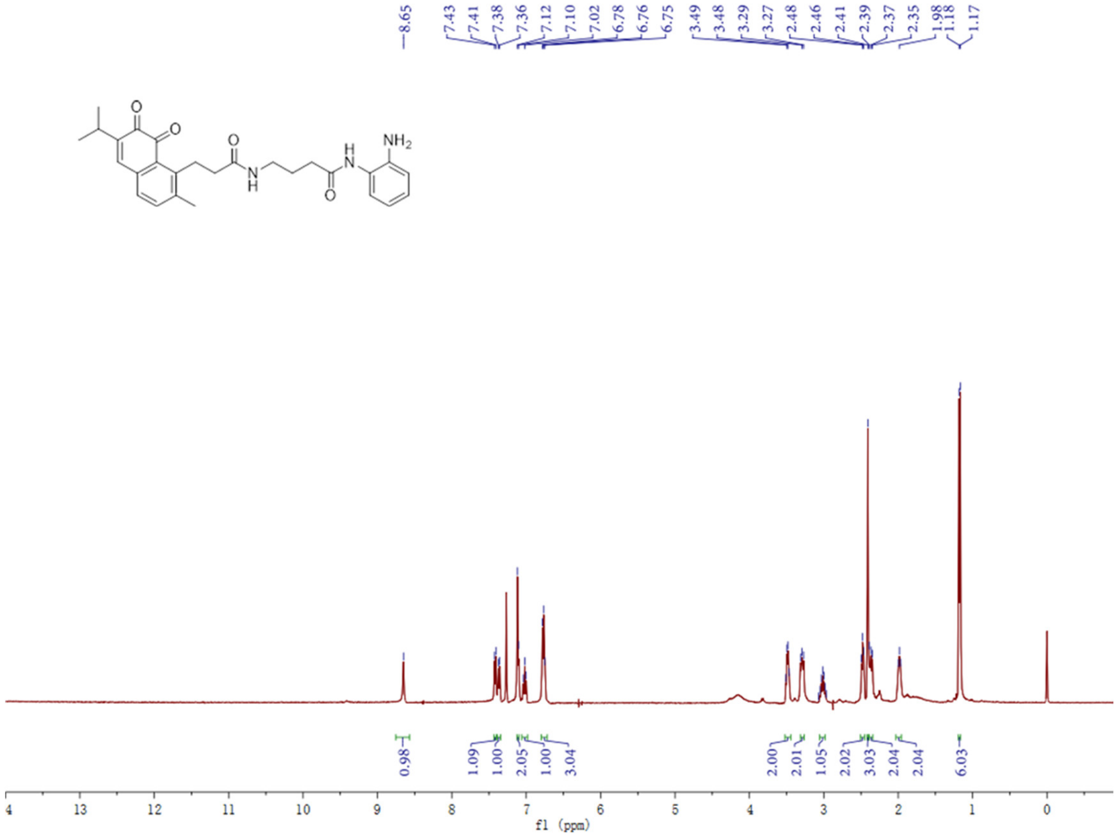

^{1}H NMR spectrum of compound **33b**

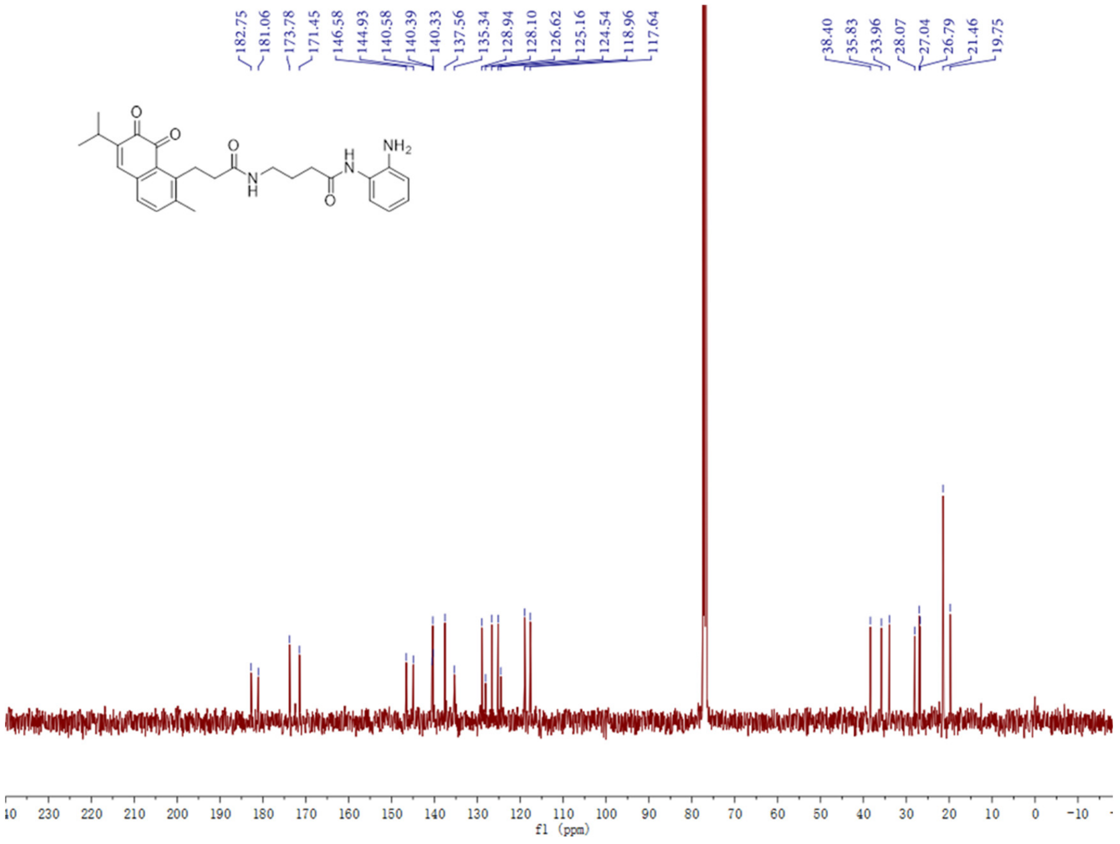

^{13}C NMR spectrum of compound **33b**

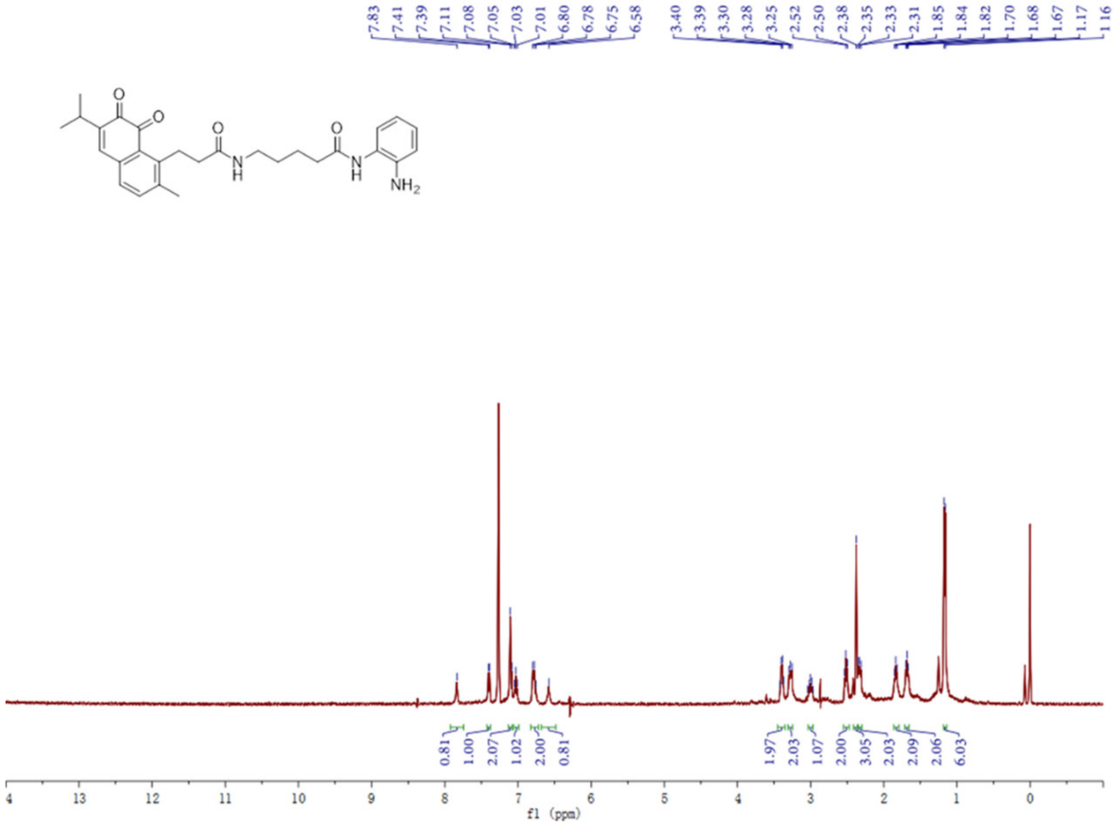

^{1}H NMR spectrum of compound **33c**

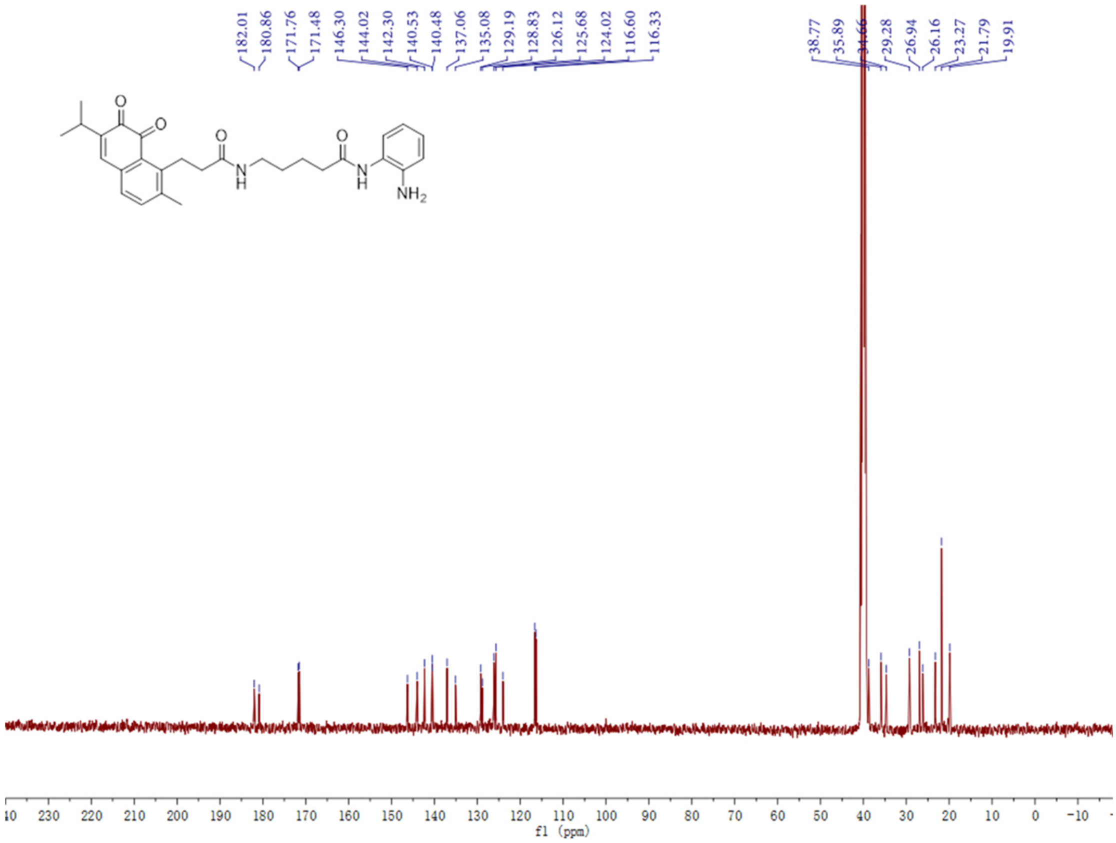

^{13}C NMR spectrum of compound **33c**

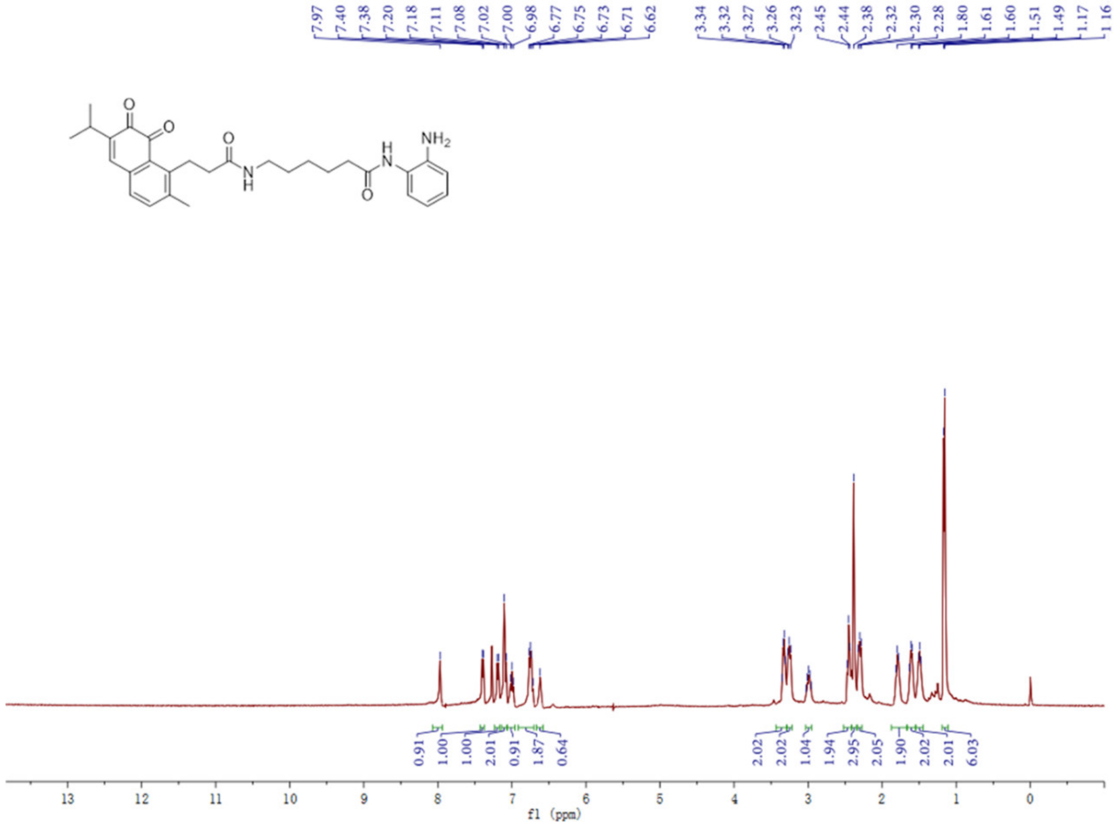

^{1}H NMR spectrum of compound **33d**

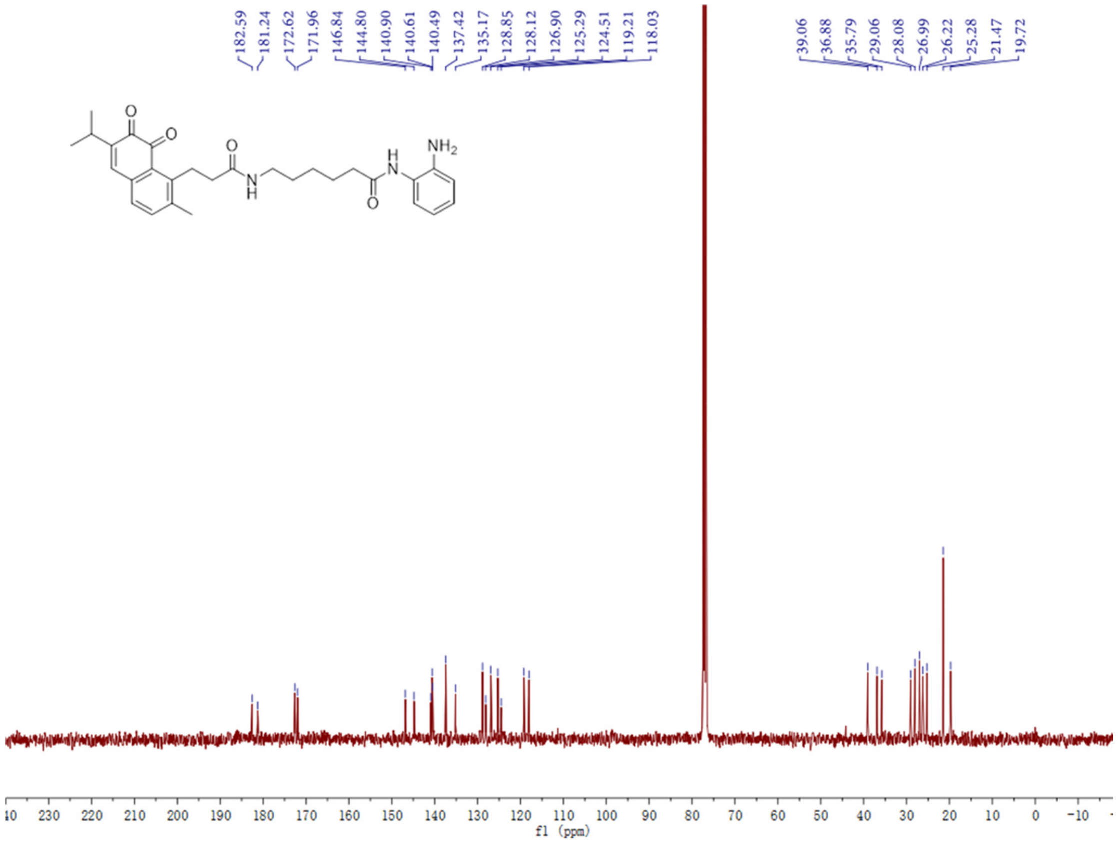

^{13}C NMR spectrum of compound **33d**

2. Software and General Procedure of QM/MM Simulation

2.1. Compilation of GROMACS with MOPAC and RM1

Protein-ligand complexes underwent QM/MM simulations by GROMACS+MOPAC+RM1 packages. The compilation of GROMACS 5.1.2 with MOPAC and RM1 method was performed by changing the AM1 atomic parameters values presented at the MOPAC source file block.f for the hydrogen, carbon, nitrogen, oxygen, phosphorus, sulfur, fluorine, chlorine, bromine and iodine atoms by RM1 parameters.[1] Then the package compilation was made according to the following procedure: (i) GROMACS (www.gromacs.org) was downloaded and extracted into the GROMACS folder; (ii) The MOPAC 7 source (openmopac.net/Downloads/Downloads.html) was downloaded and also extracted to the GROMACS folder; (iii) Gmxmop.f and decart.f were copied to the GROMACS folder in order to replace the mopac.f, moldat.f and deriv.f sources and all other codes were used to create object files with the FORTRAN 77 compiler (f77 -O3 -c *.f)[2]; (iv) All the objects (*.o) were collected into the libmopac.a library: ar rcv libmopac.a *.o and ranlib libmopac.a; (v) The libmopac.a library was moved to the GROMACS folder and the GROMACS+MOPAC+RM1 package was configured with the flags: CPPFLAGS=-DUSE_MOPAC, LIBS=-lmopac and LDFLAGS=-L$PWD.

2.2. General procedure of input file set-up in QM/MM simulation

(i) Create the gro and top files. Firstly, we create a new directory and save the out.pdb under this directory. Type command and select charmm27 forcefield and TIP3P water molecules: *gmx pdb2gmx -f out.pdb -o out.gro -p topol.top -ignh*. Then we obtain the three files: the structural file out.gro, the topology file topol.top and the position restriction file porse.itp.

(ii) Adding link atoms. Before QM/MM simulation, we should modify the gro and top files, and specify which atoms should be included in QM region and specify the QMMM parameters in mdp file. Since we broke the bond when dividing the QM region and the MM region, we need to add n linking atoms (LA) at the boundary between the QM and MM regions, so the total number of atoms in the system should increase n compared to the original. We should add the coordinates of these n atoms into the structural file out.gro, and we save the modified structural file as qmmm.gro. Then we should modify the topology file. Firstly add the LA atom types, and add [atomtypes] *LA 1 0 0 A 0 0 ; LA* to the #include statement. Note that it is very important to make sure that [atomtypes] is located after [defaults] in the expanded content. After the atomic type of LA is added, LA should also be added to the molecular type section of the topology file to keep the topology consistent with the structure file. First, we add the following to the end of the [atoms] section of the topology file: *x LA 9 QMM LA1 82 0.000 0*. The first column is the number of the LA atom in its molecular type, the second column corresponds to the atomic type in the force field, the third column is the residue number, the fourth column is the residue name, the fifth column is the atomic name, the sixth column is the atomic number, then the atomic charge, and the last column is the mass of the atom. Next we need to modify the bonding interactions section of the molecule by changing the bonding type between the QM and MM atoms to 5, indicating that the two atoms are non-interacting. Finally, we need to define the virtual site where the LA atoms are located in the topology file. By defining the virtual site, GROMACS updates the coordinates of the virtual site according to the defined constraints during the subsequent simulations. Add [virtual_site2] after [dihedrals]: *site from funct a*, where site is the number of the virtual site in the molecular structure, from means which atoms the virtual site is computed. Funct is the type of function to compute the virtual site, here is 1, a is the constraint parameter. Add the [constraints] part: *from 2 0.152*, here 0.152 is the bond length between C and N. Here the GROMACS file is completely modified. The next step is specify which atoms should be included in QM region. Type command: *gmx make_ndx -f qmmm.gro -o index.ndx*. Type: *q*. Using make_ndx, we generate a new index file (index.ndx) for the qmmm.gro, and add the following part: [QMatoms] *atomic numbers* to specify the atoms included in QM part as "QMatoms". Note that the QM atomic group also includes virtual atoms. Then, to do QMMM simulation, we specify the QMMM parameters in mdp file. The options of the QMMM parameters depends on the system.

(iii) Generate tpr file. Type command to make simulation: *gmx grompp -f *.mdp -c qmmm.gro -p topol.top -n index.ndx -o nvt.tpr -maxwarn 3*; *gmx mdrun -deffnm nvt*. If the track file and log file are updated, the simulation has run successfully.

References

1. Rocha, G.B.; Freire, R.O.; Simas, A.M.; Stewart, J.J. RM1: A reparameterization of AM1 for H, C, N, O, P, S, F, Cl, Br and I. *J. Comput. Chem.* **2006**, *27*, 1101–1111.
2. Groenhof, G.; Bouxin-Cademartory, M.; Hess, B.; De Visser, S. P.; Berendsen, H. J. C.; Olivucci, M.; Mark, A. E.; Robb, M. A. Photoactivation of the Photoactive Yellow Protein: Why Photon Absorption Triggers a Trans-to-Cis Isomerization of the Chromophore in the Protein. *J. Am. Chem. Soc.* **2004**, *126*, 4228–4233.
